# Transcriptome and DNA Methylome Reveal Insights Into Phytoplasma Infection Responses in Mulberry (*Morus multicaulis* Perr.)

**DOI:** 10.3389/fpls.2021.697702

**Published:** 2021-08-03

**Authors:** Chaorui Liu, Xiaonan Dong, Yuqi Xu, Qing Dong, Yuqi Wang, Yingping Gai, Xianling Ji

**Affiliations:** ^1^State Key Laboratory of Crop Biology, Shandong Agricultural University, Taian, China; ^2^College of Forestry, Shandong Agricultural University, Taian, China

**Keywords:** mulberry, phytoplasma, DNA methylation, transcriptome, G-type lectin S-receptor-like serine/threonine protein kinase

## Abstract

To reveal whether the response of mulberry to phytoplasma infection is associated with genome-wide DNA methylation changes, the methylome and transcriptome patterns of mulberry in response to phytoplasma infection were explored. Though the average methylation level of the infected leaves showed no significant difference from that of healthy leaves, there were 1,253 differentially methylated genes (DMGs) and 1,168 differentially expressed genes (DEGs) in the infected leaves, and 51 genes were found simultaneously to be differently methylated and expressed. It was found that the expression of G-type lectin S-receptor-like serine/threonine protein kinase gene (*Mu-GsSRK*) was increased, but its methylation level was decreased in the pathogen-infected or salicylic acid (SA)-treated leaves. Overexpression of *Mu-GsSRK* in *Arabidopsis* and in the hairy roots of mulberry enhanced transgenic plant resistance to the phytoplasma. Moreover, overexpression of *Mu-GsSRK* enhanced the expressions of pathogenesis-related protein 1, plant defensin, and cytochrome P450 protein CYP82C2 genes in transgenic plants inoculated with pathogens, which may contribute to the enhanced disease resistance against various pathogens. Finally, the DNA methylation dynamic patterns and functions of the differentially expressed and methylated genes were discussed. The results suggested that DNA methylation has important roles in mulberry responses to phytoplasma infection.

## Introduction

Phytoplasmas are cell wall-less plant pathogens of the class, *Mollicutes* (Sugio et al., 2011), and they are associated with hundreds of diseases in more than 1,000 plants in the world and cause serious losses in vegetables, fruit crops, and ornamental plants (Bertaccini and Duduk, 2009; Cao et al., 2019). Phytoplasmas colonize in the host phloem tissue, where they secrete a variety of effector molecules to affect the expression of host genes, resulting in the metabolism disorder of hosts which shows various symptoms (Christensen et al., 2005; Gai et al., 2018). Though some virulence factors of phytoplasmas have been described, how these pathogens manipulate the physiological functions of plant hosts remains unclear (Namba, 2019).

As an important epigenetic mechanism, DNA methylation is associated with many biological processes, including transcriptional silencing, gene regulation, and genomic imprinting (Ji et al., 2018; Huang et al., 2019). It was reported that DNA methylation plays important roles in regulating responses to biotic stresses in plants, and evidence showed that DNA methylation levels were altered in potato (*Solanum tuberosum*), *Arabidopsis thaliana*, and tomato (*S. lycopersicum*) plants infected by pathogenic fungi, bacteria, or virus (Pavet et al., 2006; Torchetti et al., 2016; Zhu et al., 2016; De Palma et al., 2019). Some studies also showed that phytoplasmas were involved in the methylation changes of host genes. In *Candidatus Phytoplasma aurantifolia*-infected periwinkle (*Catharanthus roseus*) and tomato plants, sterol-C-methyltransferase and some methylase and demethylase genes were downregulated (Jagoueix-Eveillard et al., 2001), and it was found that DNA methylation was involved in the epigenetic regulation of SlDEFICIENS (Ahmad et al., 2012). In addition, it was shown that demethylation of some genes associated with floral development were inhibited in tomato plants infected by phytoplasmas (Pracros et al., 2006). It was also revealed that there were different DNA methylation marks between spontaneous and cultivar-dependent recovery and healthy leaves, occurring within the promoters of genes involved in secondary metabolism and photosynthesis (Pagliarani et al., 2020). Moreover, it has been shown that the global DNA methylation level was reduced in the phytoplasma*-*infected *Paulownia* seedlings (Cao et al., 2014). Taken together, these studies suggested that DNA methylation may play an important role in regulating gene expression in phytoplasma-infected plants.

Mulberry trees are planted all over the world and have been used to feed silkworms for about 5,000 years. Mulberry is not only the sole feed tree for silkworms, but it is also a nutritionally and economically important fruit tree, and mulberry fruits have long been used as edible fruits and traditional medicines. Mulberry plants are susceptible to dwarf disease associated with the presence of phytoplasma belonging to the subgroup, 16SrI-B (aster yellows) (Gai et al., 2014), and when faced with phytoplasma infection, they will activate radical changes of gene expression (Gai et al., 2018). Phytoplasmas are introduced into mulberry phloem by vector insects, parasitize in the sieve elements of phloem, and directly contact the cytoplasm of sieve elements and companion cells (Sugio et al., 2011). In the process of mulberry–phytoplasma interaction, the sieve elements and companion cells can not only perceive phytoplasma invasion and initiate local defense responses but can also transmit the infection signal over long distances and activate the response at the whole-plant level (Henry et al., 2013; Gai et al., 2018). Previous studies have shown that phytoplasma infection can cause differential expression of some genes involved in signal transduction in plants (Hren et al., 2009; Gai et al., 2018; Liu et al., 2019). Since gene transcriptional reprogramming may be controlled by epigenetic modifications triggered by pathogen challenges, the infection of phytoplasma may cause the differential expression of some genes involved in signal transduction mediated by DNA methylation in plants. However, the gene expression changes associated with phytoplasma infection and the molecular mechanism at DNA methylation level are still not well understood. In this study, methylation-dependent restriction site-associated DNA sequencing (MethylRAD-Seq) which allows for *de novo* (reference-free) methylation analysis can be applied to both model plants and non-eschatological plants, and it is performed to assess genome-wide DNA methylation patterns and to identify the differentially methylated genes (DMGs) involved in response to phytoplasma infection. The information provided will facilitate the elucidation of the epigenetic mechanisms underlying the responses of mulberry to phytoplasma infection, and also provide important clues for further studying the disease resistance genes for mulberry breeding.

## Materials and Methods

### Plant Materials and Growth Conditions

Mulberry (*Morus multicaulis*) cutting seedlings were inoculated with phytoplasmas by being grafted with the scions collected from phytoplasma-infected mulberry trees Gai et al. (2018), and the cutting seedlings derived from the same mother tree were grafted with healthy scions and used as controls. The grafted mulberry seedlings were cultivated in a greenhouse under light/dark regime (16 h light/8 h dark) at 24°C with 50–60% humidity. The grafted mulberry seedlings showing Witches' broom disease symptoms were used to detect phytoplasma by PCR amplification of the 16S rRNA gene of phytoplasma using a universal primer set (Forward primer: 5′ TAAAAGACCTAGCAATAGG 3′; Reverse primer: 5′ CAATCCGAACTGAGACTGT 3′) (Ji et al., 2009). The sixth leaves from the top of the grafted healthy and phytoplasma-infected shoots were used for subsequent experiments, and three phytoplasma-infected and healthy plants were used as independent biological replicates for RNA sequencing (RNAseq) and MethylRAD sequencing analyses. *A. thaliana* (Col-0) and *Nicotiana benthamiana* seedlings were cultivated in the greenhouse under light/dark regime (16 h light/8 h dark) at 22 and 24°C, respectively, with 50–60% humidity.

### Transcriptome Analysis

Total RNA was extracted using a TRIzol reagent (Invitrogen Corporation, CA, United States), and the poly-A-containing messenger RNAs (mRNAs) were purified using beads with Oligo (dT). The purified mRNAs were used as templates to synthesize complementary DNAs (cDNAs) which were then added to a single “A” base to their ends. These cDNA fragments were subsequently connected with sequencing adapters and then were purified and underwent PCR amplification to create cDNA libraries. Transcriptome sequencing was conducted on the Illumina HiSeq™ 2000S platform, Illumina, San Diego, CA, United States. Raw reads were cleaned and assembled into unigenes and then the assembled unigenes were annotated by BLASTn searches against the *M. notabilis* genome (https://www.ncbi.nlm.nih.gov/genome/?term=Morus). The expressions of genes were calculated using the reads per kilobase million (RPKM), and if the expression value of a gene changed more than two times (*P* ≤ 0.05), and the false discovery rate (FDR) was <0.01 between infected and healthy samples, it was designated as a significantly differentially expressed gene (DEG). The identification of DEGs was performed using DESeq software (Zenoni et al., 2010). Three biological replicate experiments for healthy and infected samples and three technical replicates per biological experiment were performed.

### MethylRAD Sequencing and Relative Quantification of DNA Methylation Levels

Genomic DNA was extracted from the infected and healthy mulberry leaves using the cetyltrimethyl ammonium bromide method (Clarke, 2009) and then was digested with FspEI. The digestion productions were added with adaptor primers and amplified through PCR to create the MethylRAD libraries which were subjected to sequencing on an Illumina HiSeq2000 sequencer, Illumina, San Diego, CA, United States. Input sequencing data were cleaned and then subjected to Pair-End sequencing on a HiSeq X Ten platform. After mapping the paired-end sequencing reads, the MethylRAD sequencing data were cleaned to obtain high quality reads which were mapped to the reference CCGG/CCWGG sites built with the FspEI sites extracted from the *M. notabilis* genome using the SOAP program. A site with the sequencing depth of not <3 was determined as a reliable methylation site, and the RPKM was used to determine the relative DNA methylation levels of each site. If the methylation level of a methylation site changed more than two times (*P* ≤ 0.05) between the infected and healthy samples, it was designated as a significantly differentially methylated site. Then the methylation levels of sites that were localized in the gene regions were summed to evaluate the DNA methylation levels of the genes using the R package edge R (Robinson et al., 2010), and the thresholds of statistically significant difference were found to have a *p* < 0.05 and log2FC > 1.

### Gene Ontology Analysis

BlastN searches against the reference *M. notabilis* database were performed with online tools (https://morus.swu.edu.cn/morusdb/blast) to provide gene ID for the target genes. Gene ontology (GO) analysis was performed with online tools (https://morus.swu.edu.cn/morusdb/searchgo) based on the gene IDs obtained.

### Quantitative Real-Time PCR Analysis

Total RNA was extracted using a TRIzol reagent (Invitrogen Corporation, CA, United States) following the instructions of the manufacturer and then it was digested with DNase I. The cDNA was synthesized from 1 μg of total RNA extracted using oligo (dT)_18_ primers (Invitrogen Corporation, CA, United States) with reverse transcriptase M-MLV (Promega Corporation, Madison, WI, United States) in 20 ml reactions. The qRT-PCR was performed on the CFX96TM Real-time System (Bio-Rad Laboratories, CA, United States) according to the protocol of the kit (SYBR Premix Ex Taq™) with 20 μl of PCR mix. The PCR mix contains 2 μl of synthesized cDNA, 10 μl of 2 × SYBR Premix Ex Taq, 200 nmol l^−1^ of gene-specific primers, and 0.4 μl of 50 × ROX Reference Dye II. The PCR mix was preheated to 95°C for 30 s followed by 39 cycles of 95°C for 10 s and 58°C for 30 s. Melting curve analysis was conducted from 65°C up to 95°C followed by a 0.5°C incremental ramp to validate product specificity. The two genes, *EF1-a* and *ACTIN*, were used as endogenous controls to quantify the gene expression levels by the 2^−ΔΔCT^ method (Livak and Schmittgen, 2001). All samples were assayed in at least three biological replicates, and three technical replicates were run for each of them. The primers used for quantitative real-time PCR (qRT-PCR) are given in Supplementary Table 1.

### DNA Methylation Analysis by Semi-Quantitative PCR

Since the restriction endonuclease, FspEI can recognize 5-methylcytosine (5-mC) and 5-hydroxymethylcytosine (5-hmC) in the CmC and mCDS sites in genomic DNA, the genomic DNA double-strand can be cleaved by FspEI at CCGG and CCWGG methylated sites. The extracted genomic DNA was digested with FspEI, and then the digested DNA was used as a template to perform 28 cycles of PCR amplification. The genomic DNA without being digested was also used as a template to perform 28 cycles of PCR amplification using the same primers and PCR conditions. All the PCR experiments were repeated at least three times. The primers used for semi-quantitative PCR are listed in Supplementary Table 1.

### Gene Cloning and Phylogenetic Analysis

The isolated RNA was used to synthesize cDNA using the reverse transcriptase, M-MLV (Promega). The specific primers (Supplementary Table 2) used for PCR amplifications were designed based on the nucleotide sequence of the gene available from the *M. notabilis* genome (https://www.ncbi.nlm.nih.gov/genome/?term=Morus) database; the PCR products were separated by electrophoresis and the target DNA fragment was recovered and subcloned into the pMD18-T vector (Invitrogen). After transformation into DH5α, the positive clones were identified and selected for further sequencing. The multiple alignments of the deduced amino acid sequences with the sequences from other plants were conducted using the DNAMAN program. SignalP-5.0 Server (http://www.cbs.dtu.dk/services/SignalP/) was used with the default parameters to predict signal peptides, and TargetP-2.0 Server (http://www.cbs.dtu.dk/services/TargetP/) was used to predict the subcellular localization of proteins. Putative conserved domains were detected using the online NCBI program (https://www.ncbi.nlm.nih.gov/Structure/cdd/wrpsb.cgi). A phylogenetic tree was generated using the MEGA program by the neighbor-joining method, and bootstrapping was run 1,000 times. The three-dimensional (3D) structure of the protein was generated by the SWISS-MODEL pipeline.

### Subcellular Localization

In order to elucidate its subcellular localization, the fusion gene of Mu-GsSRK (GenBank: MN364943.1) and the green fluorescent protein gene (*GFP*) under the control of 35S was generated and cloned into the binary vector, pBI121 to produce the 35S::Mu-GsSRK-GFP expression vector. The *N. benthamiana* leaf epidermal cells were infiltrated with *Agrobacteria*, containing the vector with an optical density of 0.4. After filtration for about 48–72 h, small sections of the infiltrated leaves were excised and mounted in water. Confocal imaging was performed using a Bio-Rad MRC1024 confocal laser scanning microscope (Bio-Rad Microscience Ltd., Manchester, United Kingdom).

### Promoter Activity Analysis

The promoter of *Mu-GsSRK* (designed as *pMu-GsSRK*) was obtained with specific primers (Supplementary Table 2), and designed based on the nucleotide sequence of the gene available from the *M. notabilis* genome and used to create the promoter expression vector, *pMu-GsSRK*::*GUS* by replacing the 35S promoter and fusing it with the *GUS* gene. The *pMu-GsSRK*::*GUS* vector was then introduced into the *Agrobacterium tumefaciens* strain, GV3101 which was used to infiltrate *N. benthamiana* leaves as described previously (Arpat et al., 2012), and β-glucuronidase (GUS) expression in the infiltrated tobacco leaves was assessed by histochemical staining (Jefferson et al., 1987).

### Production of Transgenic Arabidopsis Lines

The coding region of the *Mu-GsSRK* gene was amplified and ligated into binary plasmid vector, pBI121 under the control of the 35S promoter. Then, the constructed transgenic plant expression vectors were introduced into *the A. tumefaciens* strain, GV3101 which was used to transform the wild-type (WT) Arabidopsis plants with the floral dip method (Harrison et al., 2006). Transgenic seeds were selected on selection plates [Murashige and Skoog (MS) media supplemented with 50 mg l^−1^ of kanamycin], and the T3 generation seeds from independent transgenic lines were used for further functional studies.

### Plant Treatment

Jasmonate (JA) and salicylic acid (SA) treatments were conducted by spraying 100 μmol l^−1^ of JA or 5 mmol l^−1^ of SA solution onto the adaxial surface of mulberry leaves. *Pseudomonas syringae* pv. *mori* inoculation was performed by spraying the bacterial suspension (10^8^ CFU ml^−1^) onto the adaxial surface of young leaves of mulberry. As for *Colletotrichum dematium* inoculation, it was conducted by spraying the conidial suspension (2.5 × 10^6^ ml^−1^ of conidia) onto the adaxial surface of mulberry leaves. The leaves sprayed with sterilized water were used as controls. All of the inoculated and control mulberry seedlings were incubated in a glass chamber for 48 h to maintain sufficient humidity. *P. syringae* pv. tomato DC3000 (*Pst* DC3000) inoculation was performed by injecting 50 μl of *Pst* DC3000 (10^5^ CFU ml^−1^) bacterial suspensions into the rosette leaves of 4-week-old Arabidopsis plants. The rosette leaves were detached and inoculated with *Botrytis cinerea* or *Phytophthora capsici* using 2-mm-diameter mycelium plugs taken from the actively growing strain colonies. The inoculated leaves were placed in covered Petri dishes to maintain high humidity, and disease symptoms were evaluated by determining the diameter of the lesions 1 week after inoculation. Three biological replicates per treatment were assayed and three technical replicates were performed for each sample.

### Detection of Colony-Forming Units

The leaves inoculated with *Pst* DC3000 were sampled 72 h after inoculation and ground in sterile water, and the suspension was continuously diluted 10 times with sterile water and spread-plated onto King's B medium. Colonies were counted after 48 h of incubation. All the experiments were conducted at least three times.

### Production of Hairy Root Transgenic Mulberry Plants

The *Mu-GsSRK* gene was cloned into the vector, pROK2 under the control of the 35S promoter to produce 35S::Mu-GsSRK. The pROK2 vector containing 35S::Mu-GsSRK was introduced into *A. rhizogenes* strains, K599 that are used for the transformation of mulberry seedlings. Gui sang You 62 mulberry seeds were sown in plastic pots containing vermiculite medium and watered with MS medium and grown in an artificial growth chamber at 25°C with a photoperiod of 16 h of light and 8 h of darkness. About 2 weeks after germination, the seedlings with the first two fully expanded true leaves were selected for agro infiltration. The *A. rhizogenes* strains, K599 harboring vectors, pROK2 containing 35S::Mu-GsSRK were cultured in yeast extract peptone (YEP) liquid medium containing rifampicin (20 mg l^−1^) and kanamycin (50 mg l^−1^), and then were centrifuged when its OD_600_ value reached 0.4 followed by re-suspension in 2-N-morpholino ethanesulfonic acid (MES) buffer (10 mmol l^−1^ MgCl_2_, 10 mmol l^−1^ MES-KOH and 100 μmol l^−1^ acetosyringone, pH 5.2). About 0.1 ml of *A. rhizogenes* harboring vectors pROK2 containing 35S::Mu-GsSRK suspension was injected into the junction of true leaves and cotyledons as described previously (Meng et al., 2019). At the same time, the plant injected with *A. rhizogenes* strains K599 harboring empty vectors, pROK2 were used as the control group. About 1 month later, when the hairy roots developed well, they were detected by PCR and the original roots were cut off.

### Phytoplasma Inoculation and Determination

To obtain mulberry yellow dwarf phytoplasma (16SrI-B)-infected leafhoppers (*Hishmonus sellatus*), the leafhoppers were transferred to phytoplasma–infected mulberry plants for 2 weeks. *A. thaliana* and mulberry seedlings were inoculated with mulberry phytoplasma by the sap-feeding method with phytoplasma-infected leafhoppers (Sugio et al., 2011), and the seedlings challenged with uninfected leafhoppers were used as controls. Each of the *A. thaliana* or mulberry seedling was challenged with six adult leafhoppers for 6 days. All the seedlings were incubated in a growth chamber at 26°C, 60% humidity, and under 12 h of light. Four weeks after inoculation, the DNA was extracted following the method described previously (Prince, 1993), and phytoplasma concentration in the plants was determined using real-time PCR described previously (Christensen et al., 2004). The primers and TaqMan probes used to amplify the 16S rRNA gene of phytoplasma, Arabidopsis, and mulberry are listed in Supplementary Table 2. Three biological replicates per treatment were assayed and three technical replicates were performed for each sample.

## Results

### Differential Transcriptome Analysis Between Phytoplasma-Infected and Healthy Mulberry Leaves

**Figure 1:**
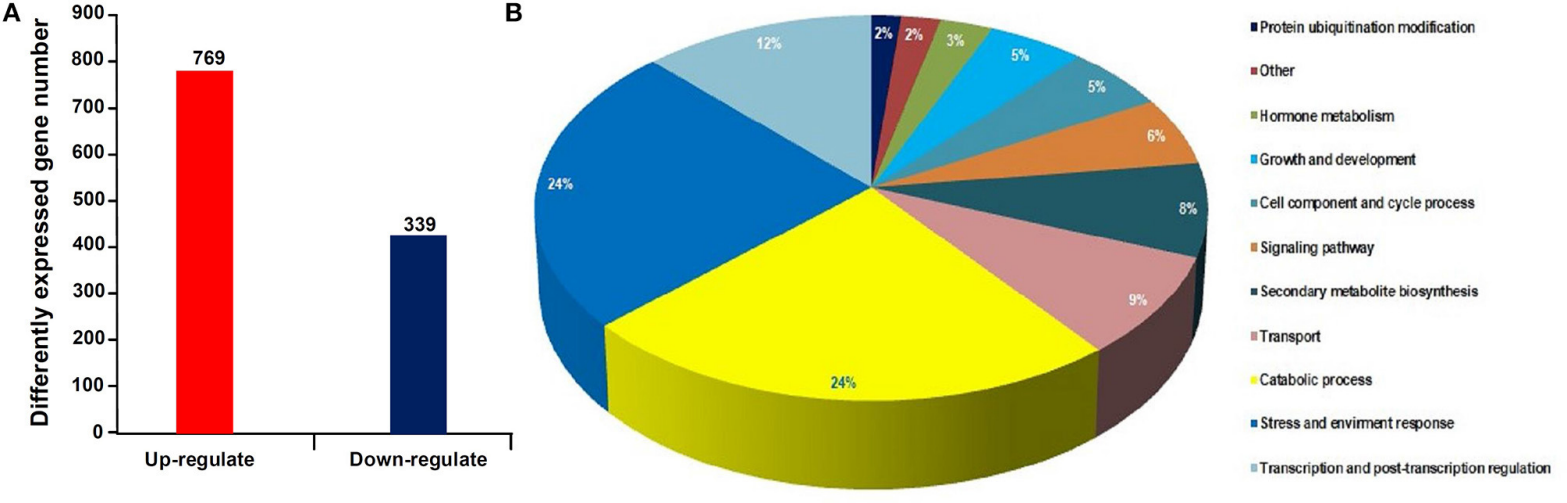
Statistical histogram of the differentially expressed genes (DEGs) and distribution percentage of them in various categories. **(A)** Number of DEGs between healthy and infected leaves. **(B)** Distribution percentage of the DEGs in various categories.

Using RNA-seq, a total of 48.9 and 48.7 million clean reads were obtained in the healthy and phytoplasma-infected mulberry (*M. multicaulis* Perr.) leaf mRNA libraries, respectively. The expression level of genes identified was calculated, and a total of 1,168 genes were found to be differentially expressed between phytoplasma-infected and healthy leaves, among which 769 genes were upregulated and 399 genes were downregulated (Figure 1A). All the detected DEGs are shown in Supplementary Table 1 and the top 40 up and downregulated ones are listed in Table 1. The GO analysis of the DEGs classified them into 11 functional categories (Figure 1B). The first category of gene was involved in stress and environment response, and the genes associated with catabolic process belonged to the second category, and the third category included the genes involved in transcription and post-transcription regulation. The other DEGs belonged to categories, such as secondary metabolite biosynthesis, signaling pathway, growth and development, cell component, and cycle process. The detailed functional GO terms of the DEGs are given in Supplementary Table 3. Interestingly, 6% of the DEGs were found to be associated with signal transduction pathways, indicating that diverse signal transduction pathways were involved in the response of mulberry to phytoplasma infection. Therefore, the regulatory networks involved in response to phytoplasma infection in mulberry plants are found to be complex.

**Table 1:** The top 40 up and downregulated genes in the infected mulberry leaves.

**Gene id**	**Normalized value**		**Fold change**	***P*-value**	**Description**
	**FPKM-HL**	**FPKM-IL**	**log2 (IL/HL)**		
LOC21398272	97.8557	0.159728	−9.407890495	3.06E-18	Glycine-rich protein 5
LOC21407207	184.53	0.431035	−8.725662659	8.57E-21	GDSL esterase/lipase LTL1
LOC21404980	272.232	1.82726	−7.716454421	1.28E-17	Putative cell wall protein
LOC21406637	20.257	0.141202	−7.572542307	2.34E-12	Protein ECERIFERUM 1
LOC21398455	3.80282	0.0331535	−6.950610547	0.0001536	Endoglucanase CX
LOC112094895	12.2097	0.161187	−6.774760712	6.39E-06	Probable protein ABIL5
LOC21388546	9.18549	0	−6.359723213	6.26E-05	Uncharacterized LOC21388546
LOC21398449	8.74903	0.112155	−6.30064398	9.37E-07	Aldehyde oxidase GLOX1
LOC21384564	10.3027	0.172242	−5.938736447	6.12E-07	Pyruvate decarboxylase 2
LOC21400381	3.48914	0.0588077	−5.902516259	0.0004514	WUSCHEL-related homeobox 1
LOC21392218	14.5559	0.277756	−5.835790145	1.35E-06	Peroxidase 19
LOC112093771	1.92941	0.0356849	−5.774760712	0.0063877	Ethylene-responsive transcription factor ERF027-like
LOC21406889	48.1223	0.847472	−5.758149749	1.55E-13	Beta-xylosidase/alpha-L-arabinofuranosidase 2
LOC112090497	2.11941	0.0340144	−5.6637294	0.0010682	Beta-glucosidase 17-like
LOC21400066	1.82909	0.0380568	−5.574462061	0.0101566	Vinorine synthase
LOC21410381	8.04636	0.20616	−5.559032021	0.0015119	Transcription factor WER
LOC21407204	432.403	9.33457	−5.521719826	2.31E-10	GDSL esterase/lipase At5g18430
LOC21400479	30.6721	0.556548	−5.490193145	8.92E-07	Leucine-rich repeat extensin-like protein 3
LOC112094128	4.28121	0.0969415	−5.446137965	0.0133982	Non-specific lipid-transfer protein 3
LOC21398811	5.90189	0.14015	−5.365648047	0.0007296	Dehydration-responsive element-binding protein 1A
LOC21406989	2.65862	0	−5.267800723	0.0036485	Homeobox protein 12
LOC21410147	8.85473	0.247533	−5.173678546	0.0002083	Ethylene-responsive transcription factor ERF109
LOC21388813	1.78319	0.0511076	−5.149156227	0.0240884	Piriformospora indica-insensitive protein 2
LOC21403374	4.10804	0.16164	−5.149156227	0.0240884	Glutaredoxin-C11
LOC21390405	38.8617	1.16881	−5.108482412	2.74E-10	Probable pectinesterase 53
LOC21410363	22.572	0.672281	−5.062900493	1.35E-09	Polygalacturonase At1g48100
LOC21397360	97.3877	2.96022	−5.060235039	1.81E-11	ABC transporter G family member 8
LOC21403148	44.9254	1.38588	−5.031733365	6.80E-11	Probable inactive leucine-rich repeat receptor-like protein kinase
LOC21410425	518.057	16.6239	−5.015647321	2.87E-10	MLP-like protein 423
LOC112094985	14.6446	0.606895	−4.97406952	0.0027701	GDSL esterase/lipase At5g45950-like
LOC112092080	5.82597	0.200124	−4.950610547	0.0002691	Glycine-rich protein 23-like
LOC21391660	0.624074	0.0242434	−4.877854205	0.0387887	Uncharacterized LOC21391660
LOC21401277	1.26248	0.0578608	−4.877854205	0.010022	Isoprene synthase, chloroplastic
LOC21399349	29.8954	1.0711	−4.864222339	2.17E-05	Uncharacterized LOC21399349
LOC21385960	1.97066	0.0731959	−4.827228132	0.0421538	Dehydration-responsive element-binding protein 2F
LOC21404198	3.95103	0.159303	−4.827228132	0.0421538	Uncharacterized LOC21404198
LOC112092755	6.40799	0.286809	−4.756838804	0.0052371	Protein GAST1-like
LOC21403098	14.7994	0.638994	−4.628682452	0.0001386	Uncharacterized LOC21403098
LOC112093610	1.2145	0.0354831	−4.584657829	0.0082983	Phospholipase D alpha 4
LOC21384131	1.88234	0.0821832	−4.543435166	0.0209278	Fatty alcohol:caffeoyl-CoA acyltransferase
LOC21412323	0.108055	5.12639	5.565089291	0.000318	Chalcone synthase 2
LOC21406747	0.303922	15.0906	5.586399797	2.55E-06	WAT1-related protein At5g07050
LOC21387954	0.0538491	2.08807	5.594836634	0.0095267	5′-nucleotidase SurE
LOC21411915	0.0303165	1.29367	5.680566508	0.0078289	receptor-like Serine/threonine-protein kinase SD1-7
LOC21396421	0.320406	19.7363	5.708047245	1.77E-08	Patatin-like protein 3
LOC21399687	0.592615	31.6888	5.789626984	3.27E-09	Blue copper protein
LOC21384783	0.0190003	0.920417	5.813016804	0.0056975	Probable LRR receptor-like serine/threonine-protein kinase
LOC21409820	0.167765	8.72595	5.813016804	0.0056975	Protein RSI-1
LOC21402368	0.39145	22.1512	5.832569602	3.63E-08	GDSL esterase/lipase EXL3
LOC21386441	0.627389	45.8209	5.857325701	2.84E-08	Miraculin
LOC21407265	0.173024	12.8049	5.949753141	5.11E-07	Ethylene-responsive transcription factor 1B
LOC21409203	0.0336459	1.90077	5.957406714	0.0039441	Uncharacterized LOC21409203
LOC21383997	0.0664015	3.96772	5.957406714	5.00E-05	G-type lectin S-receptor-like serine/threonine-protein kinase
LOC21397142	0.904786	52.6317	5.978873907	7.54E-12	Cationic peroxidase 1
LOC21406662	1.59494	95.6586	6.09016557	5.21E-14	Uncharacterized LOC21406662
LOC21408088	0	7.15596	6.139998127	0.0001645	WAT1-related protein At2g39510
LOC21406422	0.138474	8.26998	6.189580156	0.0001322	Probable glutathione S-transferase
LOC21390992	1.87224	126.612	6.200677865	7.14E-14	Asparagine synthetase
LOC112091386	0.843582	65.6859	6.250032673	3.34E-14	Feruloyl CoA ortho-hydroxylase 1-like
LOC21387901	0	2.3289	6.254388451	1.96E-07	Uncharacterized LOC21387901
LOC21410222	0.03294	2.42332	6.283907538	0.0015681	Cytochrome P450 71A1
LOC112090407	0.0500086	4.46122	6.455860221	0.0009121	Basic 7S globulin-like
LOC21406339	0.0984316	8.62995	6.471979887	0.000865	Monothiol glutaredoxin-S2
LOC21404511	0.04668	8.29125	6.519285601	2.61E-05	Gibberellin 2-beta-dioxygenase
LOC21398446	0.350073	33.1774	6.545636928	1.13E-13	Cytochrome P450 78A9
LOC21412472	16.9977	1631.78	6.591718641	0.0180694	Probable linoleate 9S-lipoxygenase 5
LOC21391507	0.156937	14.3566	6.60217861	7.71E-08	1-aminocyclopropane-1-carboxylate oxidase homolog 1
LOC21397864	0.0394556	3.97442	6.774542657	0.0002963	Probable glycosyltransferase At5g03795
LOC112092542	0.151226	16.4573	6.842247516	1.50E-07	Feruloyl CoA ortho-hydroxylase 1-like
LOC112091995	0.0271129	3.24284	6.922641296	0.0001653	Berberine bridge enzyme-like 8
LOC21384862	0.158268	29.2499	6.995077132	4.08E-08	Kirola
LOC21403962	0.0282105	3.40499	7.035409226	0.000103	Probable WRKY transcription factor 72
LOC21385733	0	15.5682	7.208945481	4.70E-05	Ribonuclease 1
LOC21400980	0	1.02752	7.342070564	1.13E-07	Uncharacterized LOC21400980
LOC21393656	11.555	2029.74	7.481901134	0.000226	Phospholipase A1-IIdelta
LOC21399608	0.0501056	9.8623	7.666627317	3.99E-06	Glucan endo-1,3-beta-glucosidase, basic isoform
LOC21393606	0.0424996	23.1696	8.242221636	1.14E-11	Probable aminotransferase TAT2
LOC21395229	0.324153	90.5203	8.2533396	1.56E-19	Pectinesterase
LOC21398841	0.0645814	58.631	9.989955408	1.59E-16	CEN-like protein 1
LOC21386143	0.0544122	91.2163	10.72411965	7.50E-21	Small heat shock protein

*FPKM, fragment per kilobase of transcript per million mapped reads. HL and IL indicate healthy and infected leaves, respectively*.

**Figure 2:**
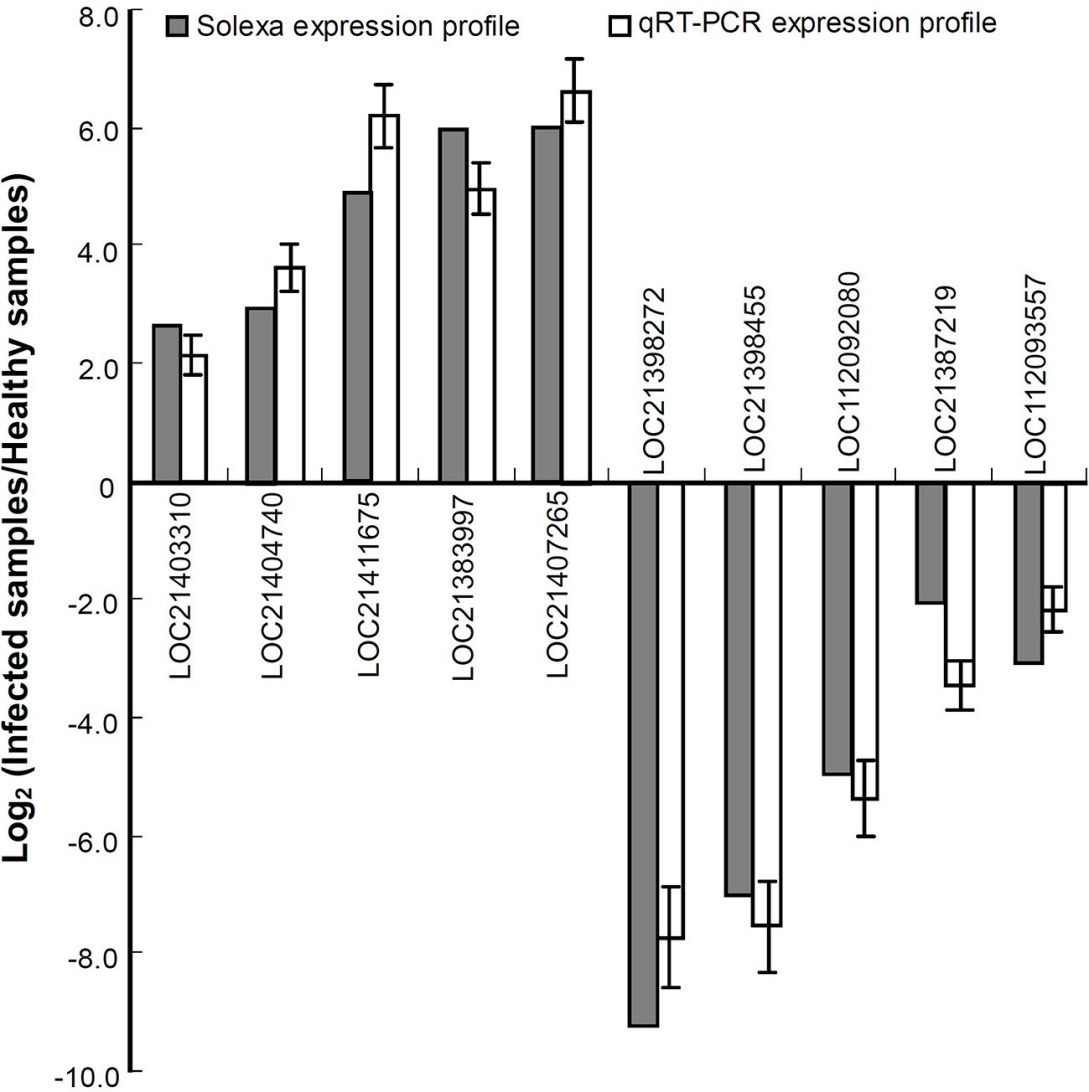
Relative expression levels of the genes analyzed by quantitative real time PCR (qRT-PCR). The relative expression levels of the genes were evaluated using the 2^−ΔΔCt^ method with *Mul-ACTIN* and *Mul-EF1-*α as reference genes. The column indicates the log_2_ ratio of phytoplasma-infected leaves/healthy leaves. Statistical values are expressed as the mean ± SD, *n* = 3 in each group.

To validate the expression profiles obtained by RNA-seq, a qRT-PCR analysis was performed for 10 genes including the upregulated and downregulated ones in response to phytoplasma infection (Figure 2). The data showed that all the selected genes exhibited similar changes in expression levels between qRT-PCR and RNA-seq results, indicating that the differential expression profiles of these genes obtained by RNA-seq are reliable.

### DNA Methylation Site Distributions

**Figure 3:**
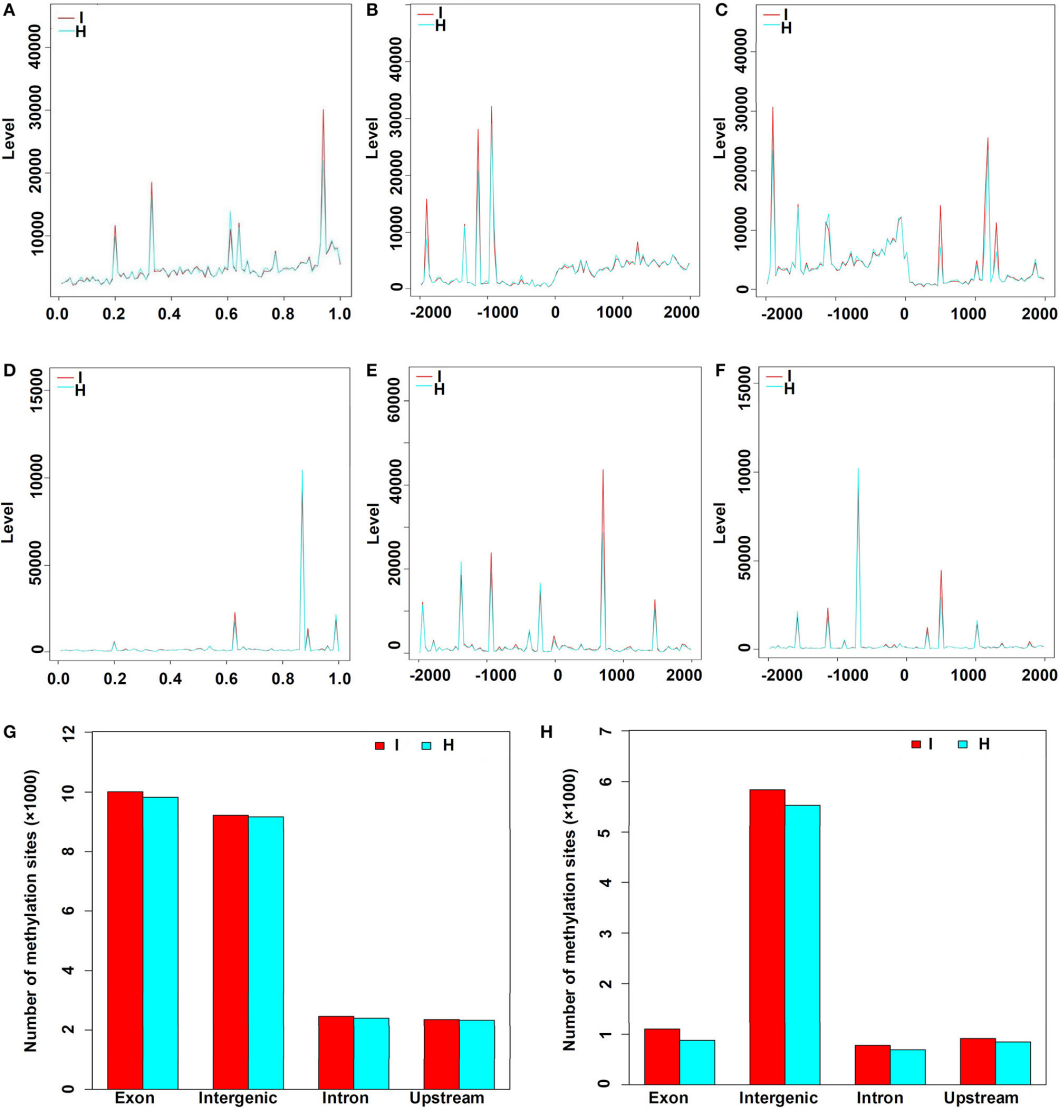
Distribution of methylation sites in different gene regions and elements. **(A)** Distribution of CCGG methylation sites in the genes. X-axis represents the relative position of the locus on the gene. **(B)** Distribution of CCGG methylation sites 2 kb upstream or downstream of the transcriptional start sites (TSS). **(C)** Distribution of CCGG methylation sites 2 kb upstream or downstream of the transcriptional termination sites (TTS). **(D)** Distribution of CCWGG methylation sites in genes. X-axis represents the relative position of the locus on the gene. **(E)** Distribution of CCWGG methylation sites 2 kb upstream or downstream of the TSS. **(F)** Distribution of CCWGG methylation sites 2 kb upstream or downstream of the TTS. **(G)** CCGG methylation sites are shown. **(H)** CCWGG methylation sites are shown. I, infected leaves; H, healthy leaves. Exon indicates the exon regions; Intergenic indicates the intergenic regions; Intron indicates the intron regions; Upstream indicates 2 kb upstream of TSS.

There were 23,056 CCGG and 8,520 CCWGG DNA methylation sites detected in the phytoplasma-infected leaves, and a total of 22,733 CCGG and 7,856 CCWGG DNA methylation sites were found in healthy leaves. When the MethyIRAD reads were aligned onto a unique locus in different regions of the genome, the genome-wide methylation pattern was obtained, and the results showed that the distributions of methylation sites in different components of the genome were differential. However, it was found that the distribution patterns of methylation sites at different elements of genomes were similar in the phytoplasma-infected and healthy mulberry leaves (Figures 3A–F). Most CCGG DNA methylation sites were mainly enriched in the exon regions followed by the intergenic, intron, and upstream regions (Figure 3G). As for the CCWGG DNA methylation sites, the major sites were mainly enriched in the intergenic regions followed by the exon, upstream, and intron regions (Figure 3H).

### DNA Methylation Levels

**Figure 4:**
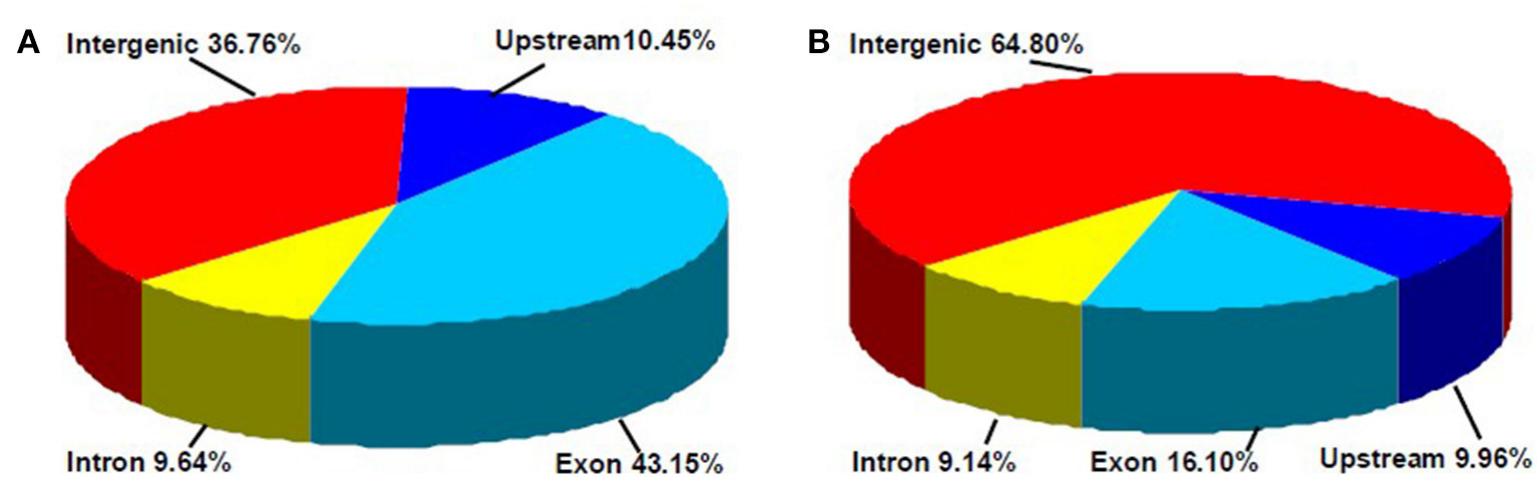
Distribution percentage of differential DNA methylation sites on different gene function components. **(A)** CCGG methylation sites are shown; **(B)** CCWGG methylation sites are shown. Exon, the regions of exon; Upstream, the upstream 2,000 bp of the transcriptional start sites (TSS); Intron, the whole introns of genes; Intergenic, the intergenic regions.

In this study, a total of 3,676 and 1,366 differentially methylated sites in CCGG and CCWGG, respectively, were found between phytoplasma-infected and healthy mulberry leaves. Most of the differentially methylated sites were found in the intergenic and exon regions, and only about 10% of the differentially methylated sites were found in the upstream and intron regions, respectively (Figure 4). The genes with differentially methylated sites in phytoplasma-infected and healthy mulberry leaves were screened and termed as DMGs. In total, 935 and 347 DMGs were identified in CCGG (Supplementary Table 4) and CCWGG sites (Supplementary Table 5), respectively. The results revealed that 511 DMGs in CCGG sites as well as 220 DMGs in CCWGG sites showed upregulation and 424 DMGs in CCGG sites as well as 127 DMGs in CCWGG sites showed downregulation in the phytoplasma-infected leaves compared to healthy leaves. Interestingly, there were 29 genes which were differentially methylated at both CCGG and CCWGG sites. These results suggested that phytoplasma infection induced the changes of methylation level of some genes.

**Figure 5:**
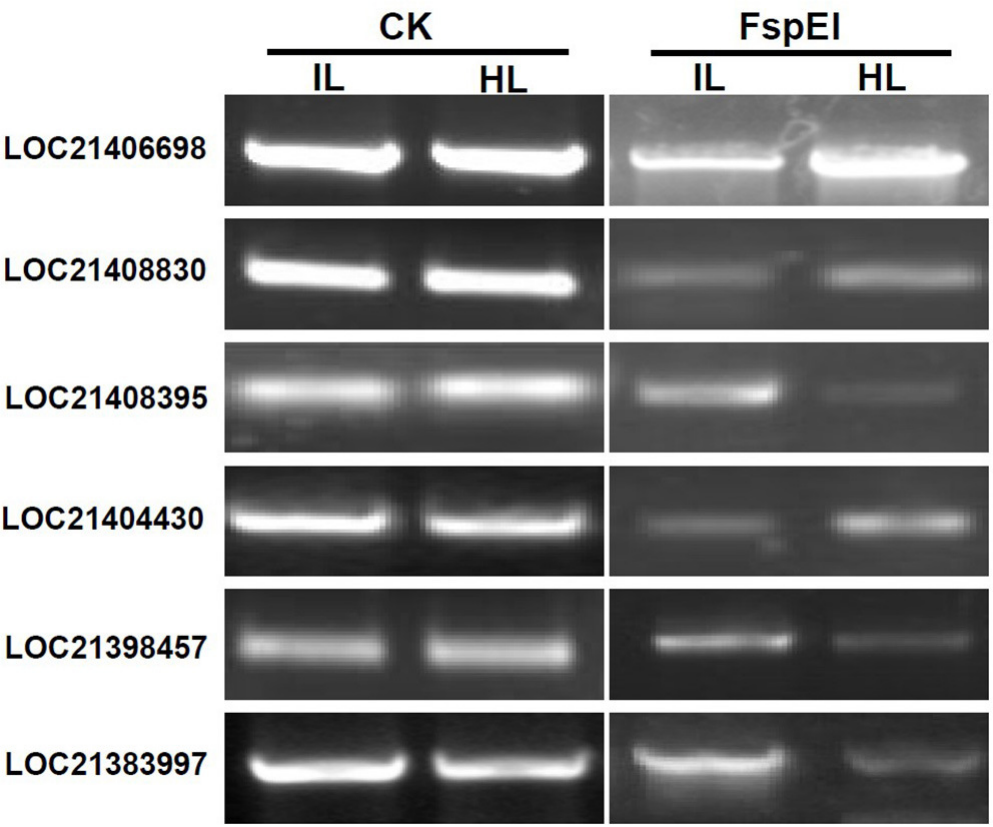
Validation of methylation levels of differentially methylated genes (DMGs) by PCR. CK, the genomic DNA without being digested with FspEI used as the template for PCR; FspEI, the genomic DNA digested with FspEI used as the template for PCR; IL, the infected leaves; HL, the healthy leaves.

To validate the results obtained with MethylRAD-seq data, six genes were selected for analysis by PCR. The genomic DNA digested with FspEI was used as the template for semi-quantitative PCR, and there were more amplification products obtained from the samples, in which the genomic DNAs were lower methylated than those from the samples in which the genomic DNAs were highly methylated at CCGG or CCWGG sites (Figure 5). The PCR results showed a high degree of consistency with the MethylRAD-seq data, indicating that the genomic DNA methylation results obtained by MethylRAD are reliable.

### Association Analysis of MethylRAD and Transcriptome Sequencing

Based on the RNA-Seq and MethylRAD sequencing data, the methylation and expression levels of the genes in the infected and healthy leaves were compared. Though a lot of genes were methylated or expressed differently, only 51 genes were differentially methylated and expressed between the infected and healthy leaves. Among these differentially expressed and methylated genes (DEMGs), there were 40 and 11 genes with CCGG and CCWGG differential methylation sites, respectively (Tables 2, 3). The correlation between DNA methylation and gene expression showed that the expression levels of 33 genes were negatively correlated with their methylation levels, while the expression levels of 18 genes were positively correlated with their methylation levels. Moreover, it was found that most of the DEMGs were differentially methylated in the exon regions, and only 5 DEMGs were differentially methylated in the intron regions and one was differentially methylated in the intergenic region (Supplementary Tables 6, 7).

**Table 2:** Association analysis of the DNA methylation levels in CCGG sites and expression levels of genes in the healthy and infected mulberry leaves.

**Gene_id**	**DNA methylation levels of genes**					**Expression levels of mRNAs**					**Description**
	**Normalized value**		**Fold-change**	***P*-value**	**Up or down**	**Normalized value**		**Fold-change**	***P*-value**	**Up or down**	
	**IL**	**HL**	**log_**2**_ (IL/HL)**			**IL**	**HL**	**Log_**2**_ (IL/HL)**			
LOC21384119	6.64	1.22	2.377247933	0.03133688	Up	10.6218	3.30881	1.696733297	0.032050552	Up	Probable acyl-activating enzyme 6
LOC21385272	57.3	28.75	1.001931754	0.010023735	Up	0.465949	4.52085	−3.07234063	0.005695407	Down	Protein NPGR1
LOC21386068	43.43	7.34	2.561160615	2.84E-07	Up	0.944625	6.06198	−2.574462061	0.005256016	Down	Proteinaceous RNase P 1, chloroplastic/mitochondrial
LOC21386721	4.22	0	5.8272901	0.019032971	Up	17.2715	96.0452	−2.450768342	0.000338362	Down	Root phototropism protein 3
LOC21387791	43.43	100.34	−1.197632275	0.000829776	Down	24.8383	5.11056	2.41804818	0.001789791	Up	Alpha-galactosidase
LOC21387864	4.83	20.8	−2.081703374	0.000736236	Down	6.71571	0.585538	3.62398298	0.000389018	Up	Glutamate receptor 2.8
LOC21388450	0	26.92	−8.469230915	9.21E-11	Down	18.8595	2.8335	2.770898828	0.000484906	Up	Remorin 4.1
LOC21389708	7.24	0	6.594271611	0.000883472	Up	9.63342	2.92863	1.734370376	0.025735456	Up	Disease resistance protein RPM1
LOC21390958	6.03	0	6.334220402	0.002947321	Up	0.694153	4.57108	−2.726142007	0.009576037	Down	LRR receptor-like serine/threonine-protein kinase RPK2
LOC21391131	21.11	9.79	1.111840833	0.037905668	Up	6.46363	0.863553	2.895734603	0.001660231	Up	Putative beta-D-xylosidase
LOC21392066	29.56	13.46	1.139403922	0.01657984	Up	24.0147	7.56114	1.682886327	0.019554235	Up	GEM-like protein 5
LOC21393642	21.71	3.66	2.548929333	6.57E-05	Up	14.2876	3.80657	1.936906262	0.014112871	Up	F-box/LRR-repeat MAX2 homolog A
LOC21393677	2.41	9.79	−1.978038631	0.021106192	Down	13.2822	174.208	−3.731234214	2.37E-06	Down	Cucumisin
LOC21393969	1.81	24.47	−3.693749899	3.50E-07	Down	11.1303	3.7693	1.622102897	0.028591888	Up	Probable serine/threonine-protein kinase WNK4
LOC21394169	1.21	6.73	−2.399293322	0.03133688	Down	3.38758	0.742111	2.40116303	0.020972852	Up	Receptor-like protein kinase 5
LOC21394538	6.03	27.53	−2.167341525	0.000108188	Down	16.638	0.407073	5.423070286	3.29E-06	Up	Heat stress transcription factor B-3
LOC21394858	25.33	1.84	3.741571181	1.60E-07	Up	24.5254	5.97396	1.911342808	0.006640769	Up	Uncharacterized LOC21394858
LOC21395936	38	10.4	1.870737662	8.77E-05	Up	7.55023	1.51213	2.185595908	0.036028134	Up	Serine/threonine-protein kinase SAPK2
LOC21396002	2.41	26.92	−3.430363844	3.31E-07	Down	69.4173	12.1258	2.535104142	0.000156284	Up	1-aminocyclopropane-1-carboxylate oxidase homolog 1
LOC21397122	3.02	18.35	−2.566597169	0.000219215	Down	7.61102	2.04864	1.952859924	0.006201517	Up	ABC transporter B family member 15
LOC21397198	2.41	14.68	−2.559283094	0.000828701	Down	23.5105	5.63271	2.089577933	0.003807881	Up	Rust resistance kinase Lr10
LOC21397218	14.48	30.59	−1.066380385	0.025670151	Down	21.892	1.09766	4.344062412	1.23E-05	Up	Homogentisate solanesyltransferase, chloroplastic
LOC21399822	8.44	1.22	2.721679835	0.007515641	Up	9.31064	29.8075	−1.661090591	0.012513308	Down	Cytochrome P450 77A4
LOC21400231	0	4.28	−5.838512532	0.019032971	Down	13.808	1.86419	2.866470008	0.007529116	Up	Cationic peroxidase 2
LOC21400376	9.05	1.84	2.26385989	0.013506003	Up	25.8076	155.143	−2.551084391	0.000280295	Down	Phosphomethylethanolamine N-methyltransferase
LOC21400775	0	9.79	−7.016905701	8.65E-05	Down	38.1527	3.69448	3.341711085	4.67E-06	Up	Aspartic proteinase nepenthesin-1
LOC21401505	4.22	23.25	−2.431079327	8.45E-05	Down	3.63616	12.0921	−1.810864686	0.022105531	Down	Endoglucanase 24
LOC21401979	60.32	24.48	1.307831037	0.000939169	Up	22.8469	86.1436	−1.910609869	0.003886471	Down	Monocopper oxidase-like protein SKU5
LOC21402514	7.24	17.74	−1.275612742	0.032805023	Down	2.45421	10.1068	−2.01987321	0.03799655	Down	Glucan endo-1,3-beta-glucosidase 5
LOC21403912	59.11	26.92	1.141625094	0.003513335	Up	14.0438	57.7734	−2.050289552	0.002115063	Down	Putative methylesterase 11, chloroplastic
LOC21406110	0	7.95	−6.719913843	0.000489065	Down	2.90947	0.153392	4.265529009	0.035230409	Up	Probable purine permease 11
LOC21407221	38	17.74	1.104566424	0.011630501	Up	35.4039	14.059	1.363421941	0.033134801	Up	Uncharacterized LOC21407221
LOC21407679	36.79	11.62	1.66492899	0.000397285	Up	15.3024	2.69869	2.52582986	0.000680367	Up	Protodermal factor 1
LOC21407908	3.02	11.01	−1.833601232	0.020788721	Down	15.3024	2.69869	2.52582986	0.000680367	Up	Heat shock cognate 70 kDa protein 2
LOC21408351	0	3.67	−5.620316002	0.036026696	Down	8.9252	1.23402	2.856855415	0.010383176	Up	CBS domain-containing protein CBSX5
LOC21410042	3.02	18.35	−2.566597169	0.000219215	Down	40.1702	1.30865	4.890219818	1.18E-08	Up	Epoxide hydrolase A
LOC21410115	13.27	29.38	−1.132484815	0.020094715	Down	1.06778	7.68425	−2.888368427	0.00713277	Down	O-acyltransferase WSD1
LOC21410270	22.92	11.01	1.061391626	0.039801044	Up	81.0559	10.3012	2.964684515	1.20E-05	Up	Cytochrome P450 71B37
LOC21410363	10.25	1.84	2.443023224	0.005647968	Up	0.672281	22.572	−5.062900493	1.35E-09	Down	Polygalacturonase At1g48100
LOC21410440	3.62	0	5.609126288	0.036026696	Up	21.5948	7.89128	1.504927856	0.037234392	Up	IAA-amino acid hydrolase ILR1-like 2

**Table 3:** Association analysis of the DNA methylation levels in CCWGG sites and expression levels of genes in the healthy and infected mulberry leaves.

**Gene_id**	**DNA methylation levels of genes**					**Expression levels of mRNAs**					**Description**
	**Normalized value**		**Fold-change**	***P*-value**	**Up or down**	**Normalized value**		**Fold-change**	***P*-value**	**Up or down**	
	**IL**	**HL**	**log_**2**_ (IL/HL)**			**IL**	**HL**	**Log_**2**_ (IL/HL)**			
LOC21383997	3	8.7	−1.5360529	0.020021665	Down	3.96772	0.0664015	5.957406714	5.00E-05	Up	G-type lectin S-receptor-like serine/threonine-protein kinase
LOC21385193	2.77	46.21	−4.041846754	1.89E-06	Down	0.460683	0	Inf	0.038255631	Up	Uncharacterized LOC21385193
LOC21385834	31.81	9.24	1.6961249	0.019332873	Up	1.14305	0.0640711	4.150051792	0.04322622	Up	Uncharacterized acetyltransferase At3g50280
LOC21386217	6.91	24.65	−1.873520354	0.014149991	Down	28.5432	82.7777	−1.540807039	0.017915134	Down	GATA transcription factor 8
LOC21386728	17.98	1.54	3.33439691	0.005616358	Up	83.5981	14.2825	2.545951498	0.000663556	Up	Pleiotropic drug resistance protein 1
LOC21397798	12.45	1.54	2.81044098	0.047691132	Up	32.6897	103.274	−1.662120382	0.02164995	Down	Alpha-xylosidase 1
LOC21405760	88.53	35.43	1.2488273	0.00565644	Up	32.0943	75.4566	−1.260241675	0.049769593	Down	Multicopper oxidase LPR1
LOC21406640	8.3	0	5.50959106	0.036026696	Up	6.21539	58.0157	−3.31628674	1.45E-06	Down	Protein ECERIFERUM 1
LOC21408202	2.76	27.73	−3.308560094	0.000697104	Down	8.81421	1.16728	2.850158208	0.000787834	Up	Non-lysosomal glucosylceramidase
LOC21408270	8.3	0	5.50959106	0.036026696	Up	6.28344	16.8424	−1.455547002	0.038863479	Down	UDP-glucuronate 4-epimerase 3
LOC21409371	8.3	0	5.50959106	0.036026696	Up	47.4971	2.87226	4.036016548	1.97E-08	Up	Cytochrome P450 84A1

**Figure 6:**
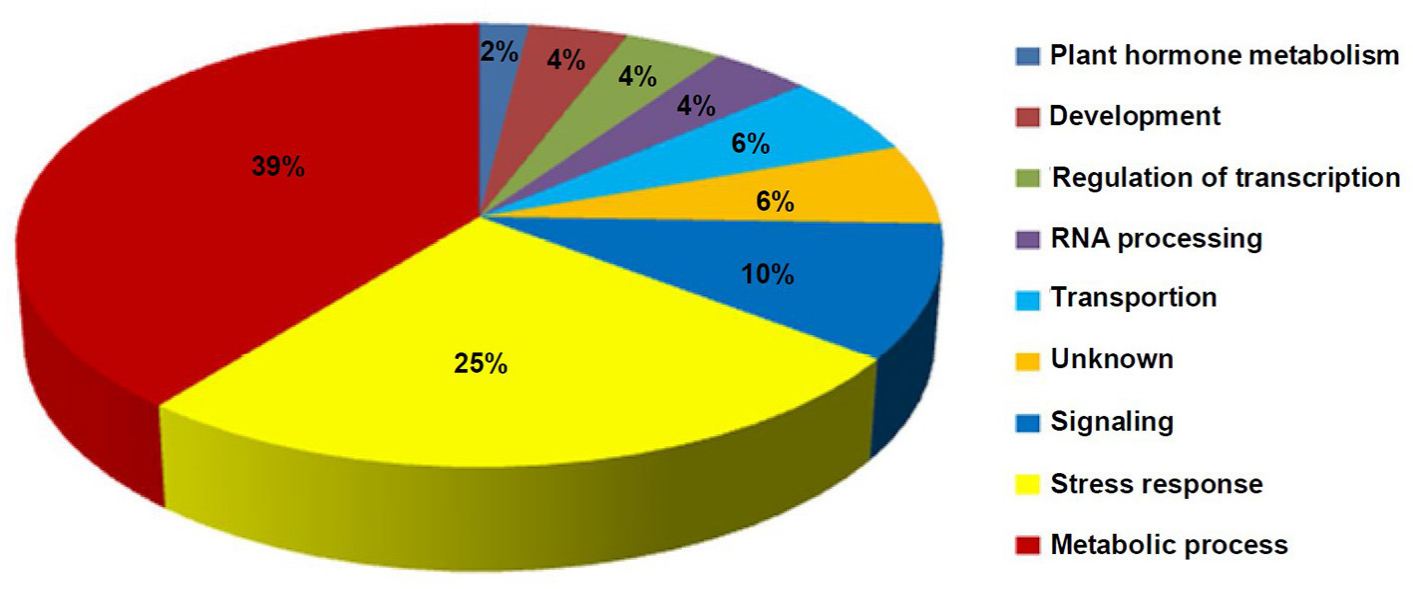
Distribution percentage of the differential DNA methylation and expressed genes in various categories.

The GO analysis of these DEMGs indicated that these genes were classified into nine functional categories (Figure 6). The first category of genes was involved in metabolism followed by the second category of genes which was associated with stress response, and the third category of genes was involved in signal transduction. The other genes were classified into transportion, RNA processing, regulation of transcription, development, and unknown categories.

### Characterization of the Phytoplasma-Responsive G-Type Lectin S-Receptor-Like Serine/Threonine Protein Kinase

**Figure 7:**
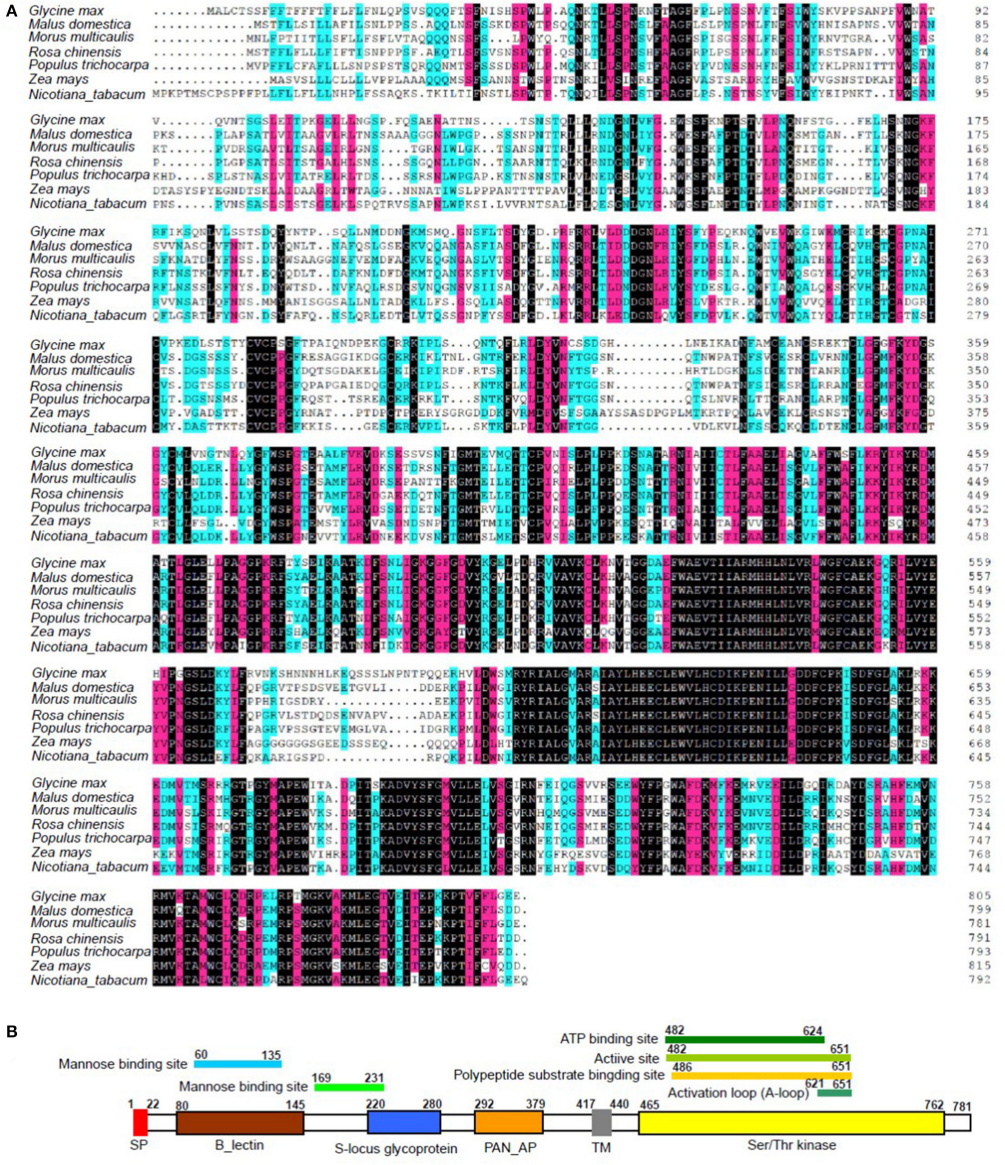
Multiple sequence alignment of Mu-GsSRK with other GsSRK proteins **(A)** and its putative conserved domains **(B)**. Amino acid residues black and red or blue-shaded are conserved and similar amino acid residues, respectively. The aligned sequences included those GsSRK proteins from *Glycine max* (XP_003529230.1), *Malus domestica* (XP_028951539.1), *Rosa chinensis* (XP_024193513.1), *Populus trichocarpa* (XP_002314767.3), *Zea mays* (PWZ52786.1), and *Nicotiana tabacum* (XP_016458326.1).

Integrated analysis showed that the expression of LOC21383997, which was annotated as *Mu-GsSRK* was increased, but its methylation level was decreased in the phytoplasma-infected leaves, and these results were confirmed by qRT-PCR (Figure 2) and semi-quantitative PCR analyses (Figure 5). The protein encoded by the *Mu-GsSRK* gene contains 781 amino acids, and its predicted molecular weight (MW) and isoelectric point (*p*I) were found to be 87.6 kDa and 6.28, respectively. Multiple sequence alignments revealed the protein-shared homology regions with GsSRKs in various species (Figure 7A). Similar to other typical G-type lectin receptor-like kinases (LecRLKs), Mu-GsSRK protein has some putative conserved domains, such as protein kinase active sites, ATP binding sites, substrate binding sites, and two activation loops. Besides these conserved kinase domains, the protein also contains a B lectin (bulb-type mannose-specific lectin) region and an S-locus-glycoprotein region at the N terminus. In addition, a plant PAN/APPLE-like domain associated with various biological functions by mediating protein–carbohydrate or protein–protein interactions was detected in the Mu-GsSRK protein (Figure 7B).

**Figure 8:**
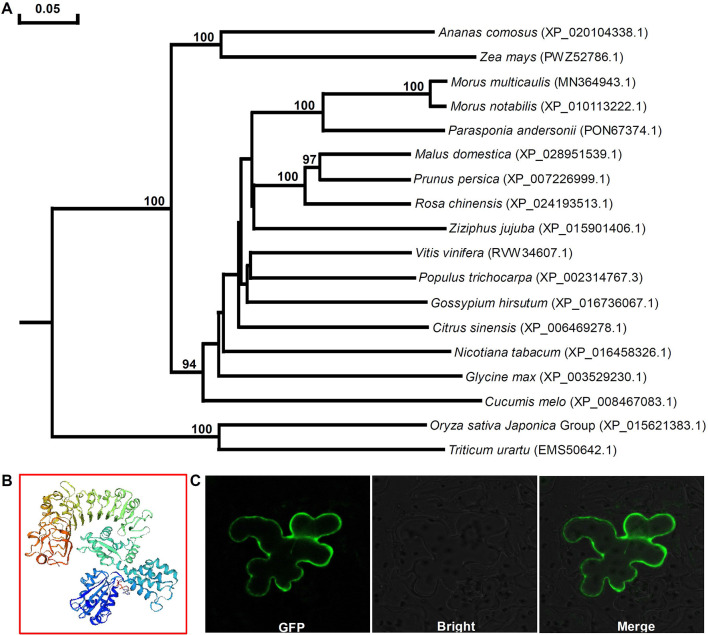
Phylogenetic analyses of GsSRK proteins from different plants and predicted three-dimensional structure and subcellular localization of Mu-GsSRK. **(A)** Phylogenetic tree was generalized using the neighbor-joining method. The numbers given on the nodes are bootstrap values, and the scale indicates genetic distance. GenBank accession numbers of the proteins are shown in the brackets. **(B)** Three-dimensional structure of Mu-GsSRK proteins was established by SWISS-MODEL. **(C)** Subcellular localization of Mu-GsSRK-GFP in *N. benthamiana* leaf epidermal cells. Mu-GsSRK-GFP fusion protein was transiently expressed in *N. benthamiana* leaf epidermal cells and visualized with a confocal laser scanning microscope (Zeiss LSM880, Zeiss, Jena, Germany). The left image shows the cell with GFP signal, and the bright-field view of the same cells is shown in the middle image. The right image indicates the overlays of the fluorescent and bright images.

Phylogenetic analysis of Mu-GsSRK and GsSRKs from other plants showed that there was closest homology between Mu-GsSRK and GsSRK in *M. notabilis* (Figure 8A). The structural properties of the Mu-GsSRK protein was predicted using SWISS–MODEL, and the result showed that the protein contained 54.93% random coil, 23.30% alpha helix, and 21.77% extended strands and had a β-barrel structure composed of a number of strands in their N-terminal lectin domains (Figure 8B). The prediction of N-terminal extension suggested that Mu-GsSRK contained an obvious signal peptide, but no obvious sublocalization sequence was detected. In order to elucidate its subcellular localization, *Mu-GsSRK* was fused to a green fluorescent protein gene and introduced into *N. benthamiana* leaves. The green fluorescent signal was detected within the plasma membrane suggesting the localization of Mu-GsSRK in the plasma membrane (Figure 8C). Therefore, as a protein kinase, Mu-GsSRK may play a role in sensing and transmitting signals arising from different biotic stress conditions.

### Expression Profile of *Mu-GsSRK*

**Figure 9:**
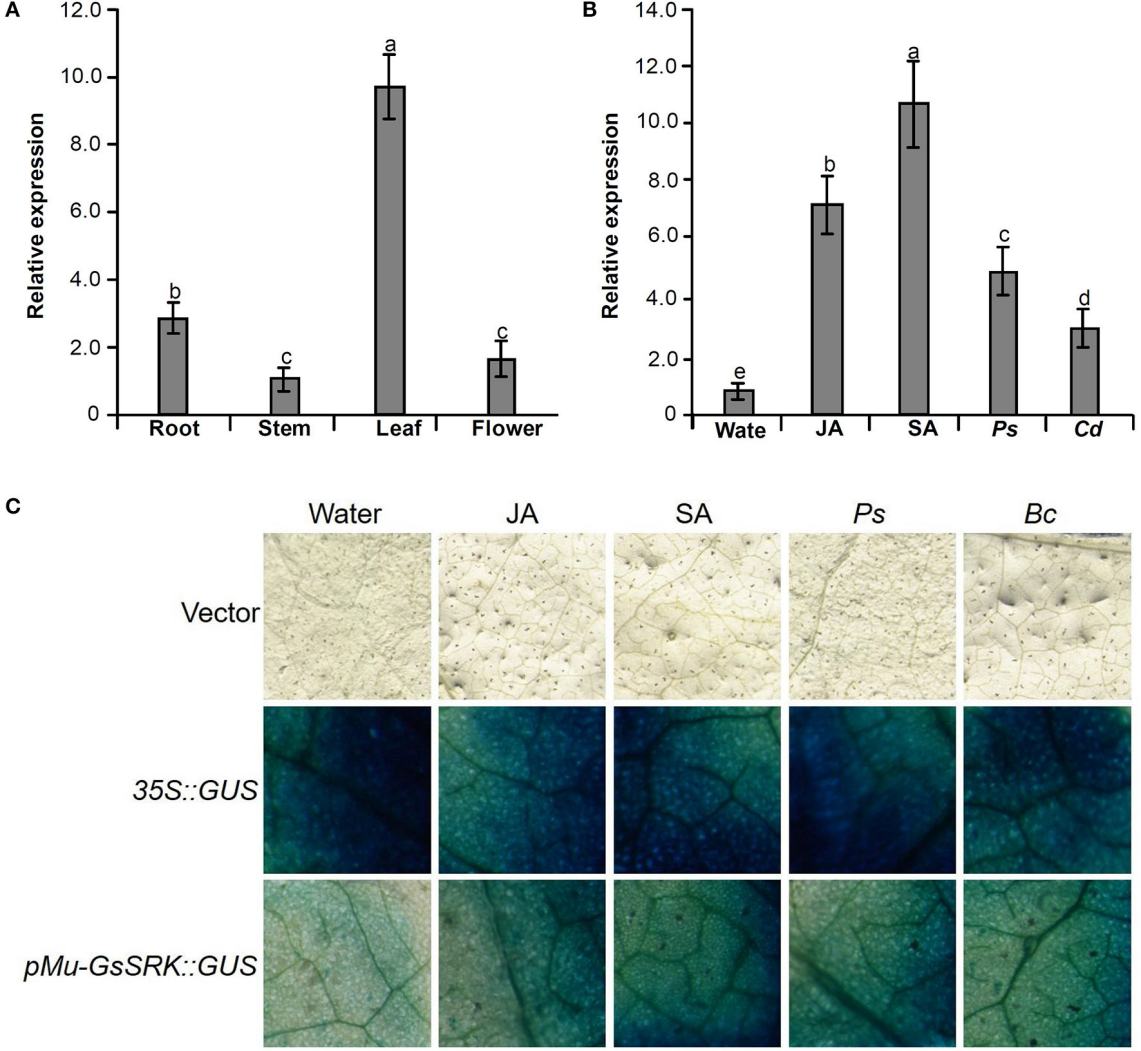
Expression pattern of the *Mu-GsSRK* gene. **(A)** Tissue expression pattern of *Mu-GsSRK*. **(B)** Induced expression pattern of *Mu-GsSRK*. *Ps* and *Cd* indicate *P. syringa*e pv. *mori* and *C. dematium*, respectively. The relative expression levels of the genes were evaluated using the 2^−ΔΔCt^ method with *Mul-actin* and *Mul-EF1-*α as reference genes. Data represent the mean values of triplicate samples ± SD. Columns with different letters above indicate significant differences at *P* < 0.05 according to Duncan's multiple range test. **(C)** Transient expression of *pMu-GsSRK::GUS* fusion in *N. benthamiana* leaves. Tobacco leaves infiltrated with transformed *Agrobacterium* were sampled at 36 and 72 h after *Pst* DC3000 and *B. cinerea* inoculation, respectively. The leaves infiltrated were sampled at 6 h after salicylic acid (SA) or jasmonic acid (JA) treatments.

To explore the function of the *Mu-GsSRK* gene, its expression pattern in different tissues and organs was analyzed by qRT-PCR. The results showed that *Mu-GsSRK* was expressed ubiquitously in the investigated parts of mulberry plants, but its expression level was higher in the leaves than that in other parts (Figure 9A). Moreover, the induced expression pattern of *Mu-GsSRK* was explored by challenging the mulberry seedlings with *P. syringae* pv. *mori* and *C. dematium* and by treating the seedlings with JA and SA, respectively. The results indicated that the expression level of *Mu-GsSRK* was increased in the leaves challenged by *P. syringae* pv. *mori* or *C. dematium*. Furthermore, it showed that the exogenous application of JA or SA enhanced the expression of *Mu-GsSRK* in the leaves (Figure 9B). In addition, *pMu-GsSRK* was fused with the GUS gene and then was transiently expressed in tobacco leaves. The results of GUS staining showed that GUS activity was induced in the leaves infected by *B. cinerea* or *Pst* DC3000, as well as in the leaves upon inoculation with JA or SA (Figure 9C). These data indicated that *Mu-GsSRK* may be associated with stress resistance, and JA and SA may modulate its expression in mulberry.

### Methylation Profile of *Mu-GsSRK*

**Figure 10:**
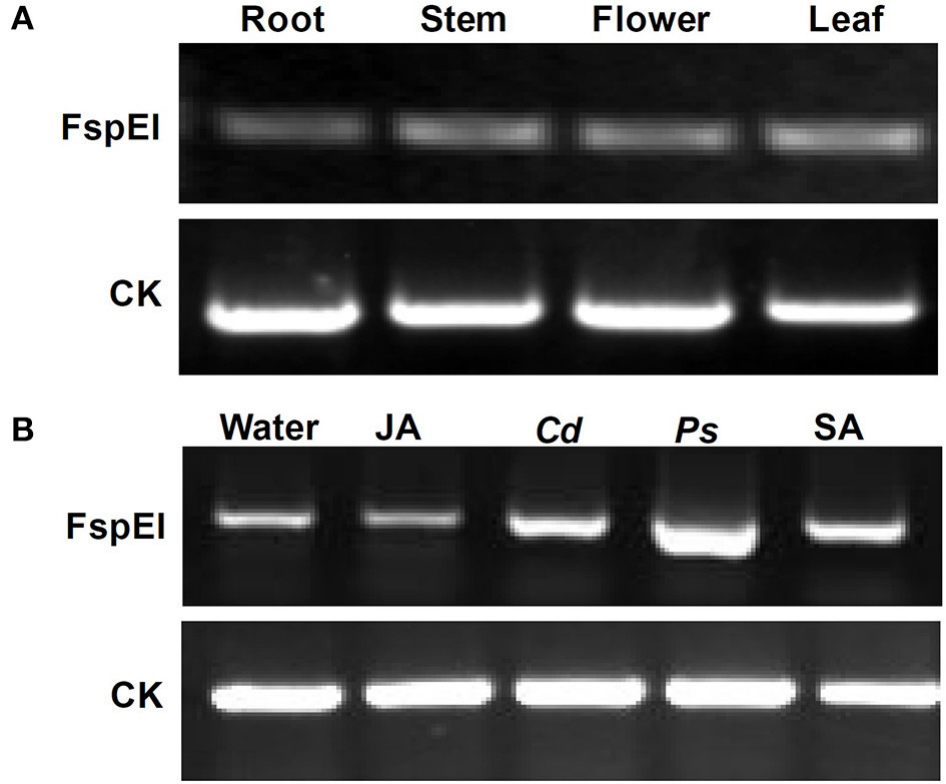
Methylation profile of *Mu-GsSRK* analyzed by PCR. Methylation level of the *Mu-GsSRK* gene in different organs detected by PCR **(A)**. Methylation level of the *Mu-GsSRK* gene in the leaves inoculated with *P. syringa*e pv. *mori* or *C. dematium* and treated with SA or JA detected by PCR **(B)**. *Ps* and *Cd* indicate *P. syringa*e pv. *mori* and *C. dematium*, respectively.

To explore whether DNA methylation was involved in the expression change of the *Mu-GsSRK* gene, genomic DNA digested with FspEI was used as the template for PCR analysis. The semi-quantitative PCR results indicated that the *Mu-GsSRK* gene was methylated at CCGG or CCWGG sites, but the methylation level of *Mu-GsSRK* did not differ significantly among the different parts of mulberry plants (Figure 10A). Therefore, the expression difference of *Mu-GsSRK* in the different parts is not caused by DNA methylation. However, the PCR results indicated that the methylation level of the *Mu-GsSRK* gene was reduced significantly in the leaves inoculated with *P. syringa*e pv. *mori* or *C. dematium* or treated with SA, but its DNA methylation level may not be affected by JA treatment. So, the expression change of *Mu-GsSRK* in the leaves inoculated with *Pst* DC3000 or *C. dematium* or treated with SA may be also caused by its methylation level change (Figure 10B).

### Overexpression of *Mu-GsSRK* in *A. thaliana* Enhances Disease Resistance

**Figure 11:**
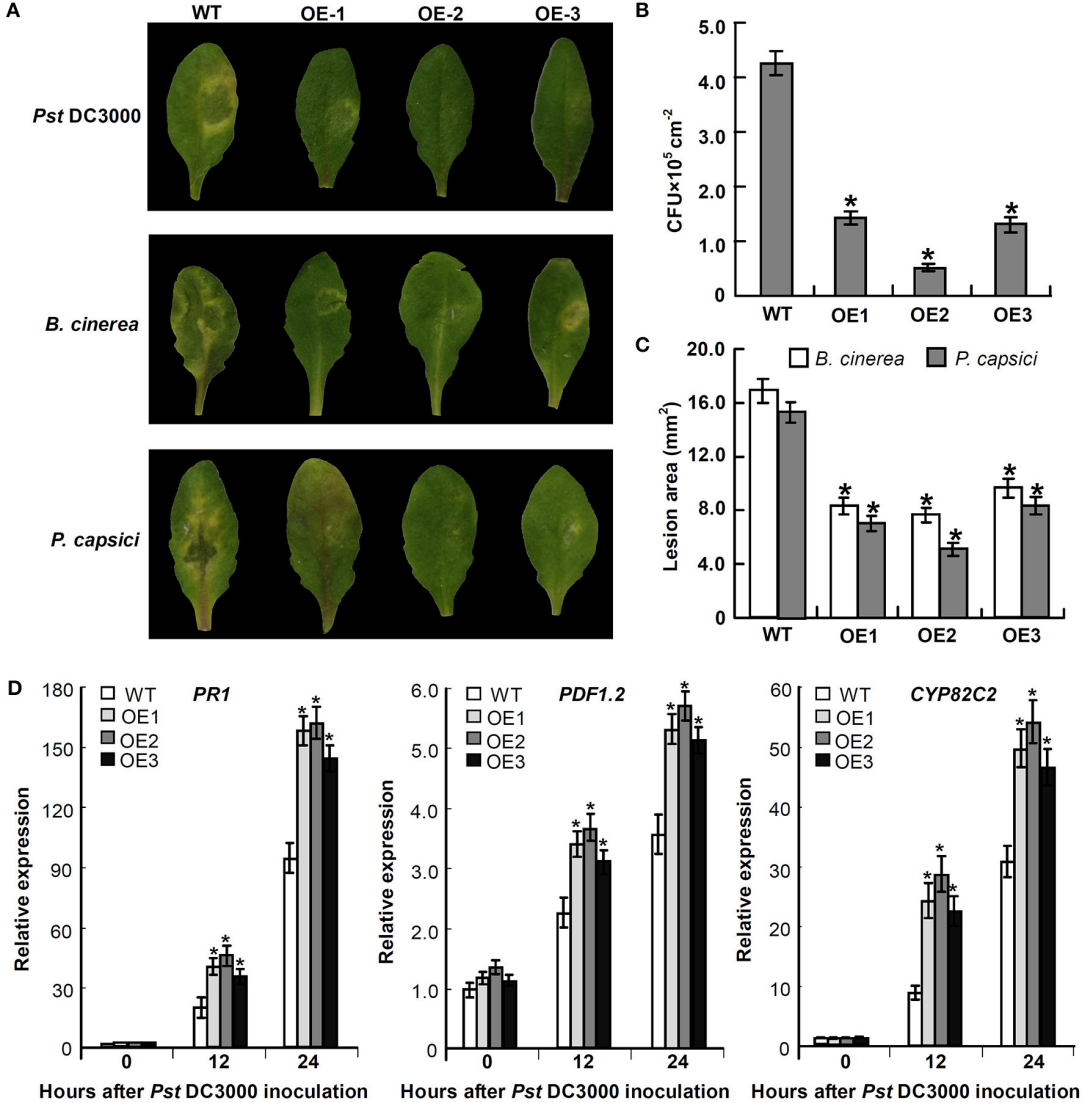
Transgenic *Mu-GsSRK* gene *A. thaliana* plants that are resistant to *Pst* DC 3000, *B. cinerea*, and *P. capsici*. **(A)** Disease symptoms observed on the leaves at 3 days post-inoculation (DPI) with *Pst* DC 3000 and 4 DPI with *B. cinerea* or *P. capsici*. **(B)** Bacterial populations of *Pst* DC 3000 strains in the inoculated leaves at 3 DPI. Values represent the mean and SD of three leaves for three independent plants. The asterisk indicates significant difference at *P* < 0.05 between WT and OE according to Student's *t*-test. **(C)** Lesion area in the leaves of WT and OE plants inoculated with *B. cinerea* and *P. capsici*. Lesion areas were measured 7 days after inoculation by determining the average lesion diameter on three leaves per line. Values represent the mean and SD of three leaves for three independent plants. The asterisk indicates significant difference at *P* < 0.05 between WT and OE according to Student's *t*-test. **(D)** Expression of *PR-1, PDF1.2*, and *CYP82C2* in *Mu-GsSRK*-overexpressing *A. thaliana* plants. The relative expression levels of the genes were evaluated using the 2^−ΔΔCt^ method with *Ath-actin* and *Ath-EF1-*α as reference genes. Data represent the mean values of triplicate samples ± SD. The relative expressions of the genes were compared with their expressions in WT without treatment, and the asterisk indicates significant difference at *P* < 0.05 between WT and OE according to Student's *t*-test. WT indicates wild-type plants and OE indicates *Mu-GsSRK*-overexpressing plants.

To explore the defensive role of *Mu-GsSRK*, wild-type (WT) and transgenic *Mu-GsSRK* plants were challenged by *Pst* DC3000. Three days post-inoculation (DPI), the WT plants showed severe disease symptoms. In contrast, there were no evident disease symptoms observed in the leaves of transgenic plants. Meanwhile, the transgenic *Mu-GsSRK A. thaliana* plants were challenged by *B. cinerea* or *Phytophthora capsici* to explore the possible role of *Mu-GsSRK* in defense against fungal pathogens. Four DPI, *B. cinerea* or *P. capsici* were successfully colonized on the inoculation leaf surface of WT plants, and obvious necrotic lesions were observed on the leaves. On the contrary, mild disease symptoms were observed on the inoculated leaf surface of *Mu-GsSRK*-overexpressing plants (Figure 11A). In addition, the bacterial populations of *Pst* DC3000 strains in the inoculated leaves were determined, and the result revealed that the population of the strains in the leaves of WT plants was significantly higher than that in the ones of *Mu-GsSRK*-overexpressing plants at 3 DPI (Figure 11B). Moreover, the lesion areas in the leaves of WT plants inoculated with *B. cinerea* and *P. capsici* were larger than those in the leaves of *Mu-GsSRK*-overexpressing plants (Figure 11C). These results indicate that the overexpression of *Mu-GsSRK* in Arabidopsis confer enhanced resistance to *Pst* DC 3000, *B. cinerea*, and *P. capsici*, and the *Mu-GsSRK* gene may play an important role in disease resistance.

To examine whether the expressions of some defense-related genes were elevated in the *Mu-GsSRK*-overexpressing *A. thaliana* plants, the expression levels of the pathogenesis-related protein 1 (PR-1) gene, plant defensin gene (*PDF1.2*), and cytochrome P450 protein CYP82C2 gene in the transgenic *Mu-GsSRK* plants were evaluated. The data showed that the expression levels of *PR-1, PDF1.2*, and *CYP82C2* genes were all very low both in the WT and transgenic *Mu-GsSRK* plants in the absence of *Pst* DC3000 inoculation, indicating that the constitutive overexpression of the *Mu-GsSRK* gene might not affect the basal expression levels of these defense-related genes. However, after *Pst* DC3000 treatment, the expression levels of *PR-1, PDF1.2*, and *CYP82C2* genes were higher in the *Mu-GsSRK*-overexpressing plants than in WT plants at 12 and 24 h post-inoculation (Figure 11D). These results demonstrated that *Mu-GsSRK* may have a positive role in the regulation of defense gene expressions, but pathogen induction is a necessary step for defense gene expression.

### Overexpression of *Mu-GsSRK* in Mulberry Enhances Resistance to Phytoplasma

**Figure 12:**
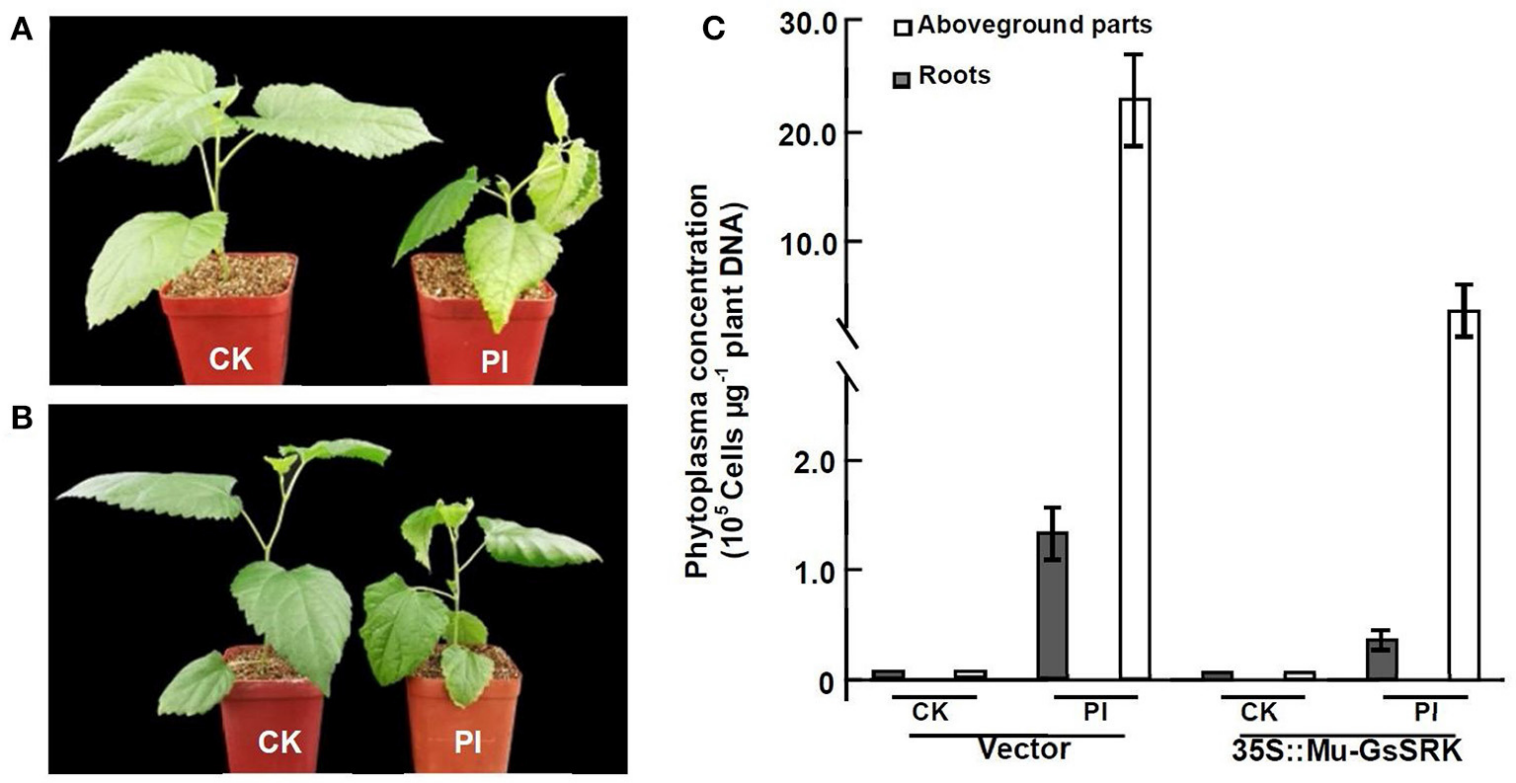
Transgenic *Mu-GsSRK* gene mulberry seedlings were resistant to phytoplasma. **(A)** Phenotypes of mulberry seedlings carrying the transgenic empty vector hairy roots challenged by phytoplasma. **(B)** Phenotypes of mulberry seedlings carrying the transgenic *Mu-GsSRK* hairy roots challenged by phytoplasma. **(C)** Phytoplasma concentration in the plants detected by real-time PCR amplification of phytoplasma 16S rDNA. Statistics values are expressed as the mean ± SD, *n* = 3 in each group. CK, plants challenged by uninfected leafhoppers. PI, plants challenged by phytoplasma-infected leafhoppers. Vector, mulberry seedlings carrying the transgenic empty vector hairy roots. 35S::Mu-GsSRK, mulberry seedlings carrying the transgenic *Mu-GsSRK* hairy roots.

Since the efficient genetic transformation and regeneration system has not yet been established in mulberry trees, in order to examine the role of *Mu-GsSRK* in the defense response to phytoplasma, transgenic *Mu-GsSRK* mulberry plants with hairy roots were generated (Supplementary Figure 1A). qRT-PCR showed that the *Mu-GsSRK* gene was constitutively highly expressed in the transgenic hairy roots (Supplementary Figure 1B). After the confirmation of transgenic *Mu-GsSRK* hairy roots, the original roots were cut off and the seedlings carrying the transgenic hairy roots were used in the follow-up of phytoplasma challenge experiments using the sap-feeding method. Four weeks post-challenge, the mulberry seedlings carrying the non-transgenic hairy roots showed severe symptoms of Witches' broom disease, such as short internodes, leaf curling, and axillary bud sprouting. Contrarily, the mulberry seedlings carrying the transgenic *Mu-GsSRK* hairy roots showed mild dwarfism symptoms and enhanced resistance to phytoplasma (Figures 12A,B). Quantitative PCR analysis showed that the concentrations of phytoplasmas in the aboveground parts and roots of the seedlings carrying the non-transgenic hairy roots were about 22.5 × 10^5^ and 1.3 × 10^5^ cells μg^−1^ plant DNA, respectively. While the concentrations of phytoplasmas in the aboveground parts and roots of the seedlings carrying the transgenic *Mu-GsSRK* hairy roots were about 6.0 × 10^5^ and 0.4 × 10^5^ cells μg^−1^ plant DNA, respectively, there was no phytoplasma detected in the plants challenged by healthy leafhoppers (Figure 12C). Therefore, the concentrations of phytoplasmas in the aboveground parts and roots of the seedlings carrying the non-transgenic hairy roots were higher than those in the seedlings carrying the transgenic *Mu-GsSRK* hairy roots, and the overexpression of the *Mu-GsSRK* gene in the hairy roots partially inhibited the growth of phytoplasmas and led to enhanced resistance to phytoplasma.

## Discussion

### DNA Dynamic Methylation Levels Were Associated With Expression Changes of the Genes Involved in Response to Phytoplasma Infection

As an important and conserved epigenetic modification, DNA methylation is associated with many important biological processes, and has been extensively studied in recent years. However, few studies have focused on mulberry, and this is the first report about the genome-wide DNA methylation profiles in mulberry. Previous reports showed that DNA methylation is not randomly distributed in genomes. In plants, the gene body regions are often highly methylated, while the transcriptional start sites (TSS) and transcriptional termination sites (TTS) mostly lack DNA methylation. Moreover, many intergenic regions also show hypermethylation; on the contrary, most promoter regions are hypomethylated (Zhang et al., 2006). The results presented here showed that DNA methylation is enriched in intergenic and gene body regions, whereas the promoter regions are also hypomethylated in mulberry genomes. In addition, it was also observed that the methylation level in exons was higher than that in introns and CCGG methylation sites (Figure 4). These results indicated that DNA methylation profiles of mulberry are analogous to those of other plants, and the methylation patterns among different plant species may be conservative. It is widely accepted that DNA methylation is a major transcription silencing pathway, which is negatively correlated with gene expression in plants (Zhang et al., 2006). However, it has also been reported that DNA methylation is positively correlated with gene expression (Lou et al., 2014). Analysis of gene expression differences between the healthy and infected leaves indicated that DNA hypermethylation is associated with activation of several genes during phytoplasma infection (Tables 2, 3). Therefore, the mechanism of gene regulation mediated by DNA methylation is very complicated and further study is necessary to elucidate it in detail.

Previous studies have shown that DNA methylation patterns can be changed when plants are infected with pathogens, and it was reported that the DNA methylation levels decreased in Paulownia plantlets infected with phytoplasma (Cao et al., 2014). However, no significant difference in the average DNA methylation level between healthy and phytoplasma-infected mulberry samples was detected (Figure 3). The transcriptome data also indicated that phytoplasma infection did not lead to significant changes in the expression of methyltransferase and demethylase in the infected samples. Although the average DNA methylation level was not changed, the methylation levels of more than 1,253 genes were changed significantly in the infected samples (Supplementary Tables 4, 5). So the methylation levels of certain genes may be dynamically regulated to control plant resistance against phytoplasma infection, but the genome was closely monitored to maintain stability. However, whether there is a link between loci-specific methylation and phytoplasma infection remains to be established.

It has been reported that DNA methylation is associated with gene expression changes in response to phytoplasma infection (Jagoueix-Eveillard et al., 2001; Pracros et al., 2006; Ahmad et al., 2012; Cao et al., 2014; Pagliarani et al., 2020). In this study, 51 DEMGs were identified in the infected leaves. Among these, there were some receptor kinase genes, such as LRR receptor-like serine/threonine-protein kinase, *Mu-GsSRK*, and serine/threonine-protein kinase SAPK2e (Tables 2, 3), which play a central role in signaling during the pathogen recognition in plants (Bouwmeester and Govers, 2009; Wang and Bouwmeester, 2017). By adjusting the methylation levels of these genes, plants can regulate their expression levels to enhance the perception of phytoplasma infection. On the other hand, phytoplasma may change the methylation level of these genes to affect their expression, thus avoiding plant perception (Zhai et al., 2010; Cao et al., 2014). In addition, there were 22 DEMGs involved in metabolic, growth and developmental processes were found in the infected leaves; the changes of these genes may disturb the normal metabolic growth and developmental processes leading to various symptoms. Although these symptoms may hinder the normal growth and development of mulberry plants, they may promote parasite multiplication of phytoplasma in the infected plants. Moreover, among these DEMGs, there were 13 genes associated with stress response (Tables 2, 3). This suggests that mulberry plants can change the expression of some disease resistance genes by altering their methylation status, so as to improve their resistance to phytoplasma. Therefore, our evidence supported that the DNA dynamic methylation levels were associated with expression changes of the genes involved in response to phytoplasma infection. Although there were many genes which were simultaneously methylated and expressed differently in the infected and healthy leaves, there is a wide variety of mechanisms that control gene expression, and DNA methylation may be the reason for the change of gene expression, but the change may be affected by other factors rather than DNA methylation.

### DNA Methylation Plays an Important Role in Regulating *Mu-GsSRK* Gene Expression in Response to Phytoplasma Infection

The lectin receptor-like kinases (LecRLKs) are categorized into three sub-classes: G-, L-, and C-type depending on the features of their N-terminal lectin domains (Vaid et al., 2012), and the Mu-GsSRK protein has some domains conserved in G-type LecRLKs (Figure 7B). So it might have similar biological functions with other G-type LecRLKs. Due to the resemblance of the extracellular domain with the lectin protein, known to bind to fungal and bacterial cell wall components, LecRLKs are hypothesized to predominantly participate in biotic stress tolerance (Wang and Bouwmeester, 2017). There were many G-type LecRLKs that have been found to be associated with the responses to pathogen infections, and some LecRLKs have been reported to confer resistance to a variety of pathogens (Chen et al., 2006; Sanabria et al., 2012; Vaid et al., 2013; Ranf et al., 2015). However, to the best of our knowledge, the current study is the first to report that G-type LecRLK genes are associated with the response to phytoplasma infection.

It was reported that some members of the LecRLK family are located on the plasma membrane and can sense signals arising from different biotic stress conditions and transmit the stress signal to the nucleus (Vaid et al., 2013). So some LecRLK family genes were proposed to play a role in mediating and strengthening the cell wall–plasma membrane (CW–PM) links and continuum which is essential for defense against pathogens (André et al., 2005; Bouwmeester et al., 2011; Singh et al., 2012; Ranf et al., 2015). Our results showed that the Mu-GsSRK protein is localized on the plasma membrane (Figure 8C), and it can significantly increase the expressions of disease resistance-related genes upon pathogenic infection (Figure 11C) and enhanced resistance to the pathogens (Figure 11A). Moreover, our results showed that the overexpression of *Mu-GsSRK* in the roots not only enhanced the roots but also the resistance of the aboveground parts to phytoplasma (Figure 12). Therefore, the Mu-GsSRK protein may be able to sense the signals arising from phytoplasma infection and transmit the signals to the nucleus and regulate the expressions of disease resistance-related genes, and the signal might be transmitted a long distance and stimulate the immune response of the whole plant.

It was reported that there was an increase in endogenous SA in the phytoplasma-infected plants (Dermastia, 2019). In addition, our results showed that the methylation level of the *Mu-GsSRK* gene was reduced significantly in the leaves treated with SA (Figure 10B). Therefore, the endogenous SA may be increased in the phytoplasma-infected mulberry leaves, which may reduce the methylation level and enhance the expression of *Mu-GsSRK*. Moreover, our results showed that the activity of the *Mu-GsSRK* promoter was induced by SA (Figure 9C). So, the activities of the *Mu-GsSRK* promoter may be induced by the increase of endogenous SA caused by phytoplasma infection and enhance the expression of the *Mu-GsSRK* gene. In response to phytoplasma infection, SA may be an important factor in the regulation of methylation and the expression level of *Mu-GsSRK* in mulberry; further study is required to elucidate the precise mechanism behind this process.

## Conclusion

In conclusion, a large number of genes with different methylation and expression levels were identified, which laid a foundation for further study on the regulation mechanism of gene expression during the response to phytoplasma infection in mulberry. Our results proved that phytoplasma infection induced changes both in the methylation and expression of *Mu-GsSRK* gene which positively regulates the resistance of plant disease. The information provided here is particularly useful to better understand the interactions between mulberry and phytoplasma.

## Data Availability Statement

The datasets generated for this study can be found in online repositories. The names of the repository/repositories and accession number(s) can be found at: PRJNA718973 (https://www.ncbi.nlm.nih.gov/sra/PRJNA718973).

## Author Contributions

XJ conceived and designed the experiments. CL, XD, YX, YW, and QD performed the experiments and analyzed the data. YG wrote the manuscript. All authors read and approved the final manuscript.

## Conflict of Interest

The authors declare that the research was conducted in the absence of any commercial or financial relationships that could be construed as a potential conflict of interest.

## Publisher's Note

All claims expressed in this article are solely those of the authors and do not necessarily represent those of their affiliated organizations, or those of the publisher, the editors and the reviewers. Any product that may be evaluated in this article, or claim that may be made by its manufacturer, is not guaranteed or endorsed by the publisher.

## Supplementary Material

Supplementary Table 1 The primers used for gene cloning.

Supplementary Table 2 The primers used for qRT-PCR and semi-quantitative PCR.

Supplementary Table 3 Differentially expressed genes (DEGs) between the healthy and infected mulberry leaves.

Supplementary Table 4 Differentially methylated genes (DMGs) in CCGG sites between the healthy and infected mulberry leaves.

Supplementary Table 5 Differentially methylated genes (DMGs) in CCWGG sites between the healthy and infected mulberry leaves.

Supplementary Table 6 Differentially methylated levels in CCGG sites in the gene regions of differentially methylated and expressed genes in the healthy and infected mulberry leaves.

Supplementary Table 7 Differentially methylated levels in CCWGG sites in the gene regions of differential methylated and expressed genes in the healthy and infected mulberry leaves.

Supplementary Figure 1 Regeneration of transgenic *Mu-GsSRK* hairy roots in mulberry seedling. **(A)** The hairy root phenotype of mulberry seedling. The arrow points to the hairy root. **(B)** Quantitative analysis of the expression levels of the *Mu-GsSRK* genes in the transgenic hairy roots. The relative expression levels of the genes were evaluated using the 2^−ΔΔCt^ method with the *Mul-actin* and *Mul-EF1-*α as reference genes. Data represent the mean values of triplicate samples ± SD. Asterisk indicates significant difference at *P* < 0.05 between WT and OE plants according to Student's *t*-test. WT, wild type plants; CK, the transgenic empty vector hairy roots; OE, the transgenic *Mu-GsSRK* hairy roots.
