# Supplementary material for: Transcriptome and DNA Methylome Reveal Insights Into Phytoplasma Infection Responses in Mulberry (Morus multicaulis Perr.)
Source: Front Plant Sci. 2021 Aug 3;12:697702. doi: 10.3389/fpls.2021.697702 (PMC8369481; doi:10.3389/fpls.2021.697702)
Supplement: Supplementary Table 1 — The primers used for gene cloning. [file Table_1.DOC]

**Table S1. The primers used for** **used for** **gene cloning.**

| **Gene names** | **Sequence (5'-3')** | |
| --- | --- | --- |
|  | **Forward primers** | **Reverse primers** |
| LOC21403310 | ACAGGATTGGTACTAGGCTG | GTAGGAACACAACCATGCAC |
| LOC21404740 | TACCTTGTGGCATTGCTGAG | TCTTGCGCTTGTGAAACTGG |
| LOC21411675 | TTAGTGATTGGAACTCCGTG | AACGACCTCTTCCTCATCTG |
| LOC21383997 | AGAGATTCTCCTACACTGAGC | AGGTGGTGCATCCTCGCAAT |
| LOC21407265 | CTACGACCAAGCGGCATTCT | TTCCTCTTGAGGGAGTGTCG |
| LOC21398272 | GAAGAACTTCCTCACCTACG | TCCACCTCCAAGTCCACCA |
| LOC21398455 | GGAGACAATCCAGCAAAGATG | AGTCGAGGTAGTGGAATCCA |
| LOC112092080 | TAGGCATGGAAGTTTCGGTG | AGCCCACCTCCACCACCTAA |
| LOC21387219 | TGCACTGAGAGCTACGCTGA | CAGAACATCACTGCAAGCAG |
| LOC112093557 | ATCTTCTGATGGACGGTGAT | CATCTGATTGAGGGCACCAT |
| LOC21406698 | ATGGAGTTGGAGGAAGAGAGA | TCATTGATGTTCCATGTCGA |
| LOC21408830 | ATGAGCATGGATGTGGCCAAG | TTAACGAAACATATTTGGCT |
| LOC21408395 | TGTTCCATGTTTCAACATCGT | CTCTTCCGAGGAGATCAACC |
| LOC21404430 | ATGGCGTTGAGTAATTGGGAA | GACGGATTTGTCATGACTACG |
| LOC21398457 | ATGGCTGGAAAAGGCGATGGT | TTAATCCACCACCTCGATCAA |
| LOC21383997 | ATGAATCTCTTCCCCACCATC | GCAAGGTTGCCCATCGATGGC |
| Pathogenesis-related protein 1 | AGACTCATACACTCTGGTGG | TGTTCGCAGCGTAGTTGTAG |
| Plant defensin gene (PDF1.2) | TTGCTGCTTTCGACGCA | TGTCCCACTTGGCTTCTCG |
| Cytochrome P450 protein CYP82C2 | TTGTATCCAGCTGGTCCTCT | GGCTCCATATAAACCCTCGG |
| Mul-actin | CACTGAGGCTCCTTTGAACCC | AGGTCGAGACGGAGAATAGCATG |
| Ath-actin | GCACCCTGTTCTTCTTACCG | AACCCTCGTAGATTGGCACA |
| Mul-EF1-α | GGGTGATTCAAGATGATGACT | TCAGTCAAGGACATCCGAAG |
| Ath-EF1-α | AGAAGGGTGCCAAATGATGAG | GGAGGGAGAGAGAAAGTCACAGA |
