# Supplementary material for: Transcriptome and DNA Methylome Reveal Insights Into Phytoplasma Infection Responses in Mulberry (Morus multicaulis Perr.)
Source: Front Plant Sci. 2021 Aug 3;12:697702. doi: 10.3389/fpls.2021.697702 (PMC8369481; doi:10.3389/fpls.2021.697702)
Supplement: Supplementary Table 2 — The primers used for qRT-PCR and semi-quantitative PCR. [file Table_2.DOC]

**Table S2. The primers used for** **qRT-PCR and semi-quantitative PCR.**

| **Primer names** | **Sequence (5'→3')** |
| --- | --- |
| Mu-GsSRK F | ATGAATCTCT TCCCCACCAT C |
| Mu-GsSRK R | CTACTCGTCACCAAGGAAAAAT |
| pMu-GsSRK F | GGAACTATTCTCGTTTTACGT |
| pMu-GsSRK R | TCCAGTAGCCAGAGACGGCGA |
| Phy-16S rRNA F | CGTACGCAAGTATGAAACTTAAAGGA |
| Phy-16S rRNA R | TCTTCGAATTAAACAACATGATCCA |
| Phy-Probe | TGACGGGACTCCGCACAAGCG |
| Ath-18S rRNA F | GACTACGTCCCTGCCCTTTG |
| Ath-18S rRNA R | AACACTTCACCGGACCATTCA |
| Ath-Probe | ACACACCGCCCGTCGCTCC |
| Mul-18S rRNA F | ACATCCCTGCCCTTTGTA |
| Mul-18S rRNA R | CCTAACACTTCATCGGACCA |
| Mul-Probe | TCGCTCCCTACCGATTGAA |
