# Supplementary material for: Transcriptome and DNA Methylome Reveal Insights Into Phytoplasma Infection Responses in Mulberry (Morus multicaulis Perr.)
Source: Front Plant Sci. 2021 Aug 3;12:697702. doi: 10.3389/fpls.2021.697702 (PMC8369481; doi:10.3389/fpls.2021.697702)
Supplement: Supplementary Table 6 — Differentially methylated levels in CCGG sites in the gene regions of differentially methylated and expressed genes in the healthy and infected mulberry leaves. [file Table_6.DOCX]

**Table S6. Differential methylated levels in CCGG sites in the gene regions of differential methylated and expressed genes in the healthy and infected mulberry leaves.**

| **Gene id** | **Sequence** | | **Gene**  **regions** | **Normalized value** | | **Fold-change** | **P-Value** | **Up/**  **down** | **Description** |
| --- | --- | --- | --- | --- | --- | --- | --- | --- | --- |
|  |  |  |  | **IL** | **HL** | **log2(IL/HL)** |  |  |  |
| LOC21397218 | | TTTGCTAATTTGAGCCGGGCAAAACGGCCCAC | Intron | 14.48 | 30.59 | 1.039227 | 0.02567 | UP | homogentisate solanesyltransferase, chloroplastic |
| LOC21384119 | | GCGACCGAGCAGGCCCGGCTCAAGGCCCGGCA | Exon | 5.43 | 0 | -6.19766 | 0.005445 | Down | probable acyl-activating enzyme 6 |
| LOC21401505 | | GGAGGATGATGACTCCGGGACTAATGTGCACA | Exon | 1.21 | 9.18 | 2.816096 | 0.004663 | UP | endoglucanase 24 |
| LOC21401505 | | CGTGTCCATATCTTCCGGTCTCTCCCAGCAAT | Exon | 0.6 | 13.46 | 4.28277 | 3.23E-05 | UP | endoglucanase 24 |
| LOC21386068 | | GTCTTTCAGTGTTACCGGTTCGTGATGGCCTC | Intron | 43.43 | 7.34 | -2.58825 | 2.84E-07 | Down | proteinaceous RNase P 1, chloroplastic/mitochondrial |
| LOC21403912 | | AGACTTGACAGGTTCCGGAATTCATTCGTCTG | Exon | 52.48 | 22.64 | -1.24649 | 0.002635 | Down | putative methylesterase 11, chloroplastic |
| LOC21407679 | | CTCCTGTGTATGCACCGGTGACACCTGCACCT | Exon | 36.79 | 11.62 | -1.69207 | 0.000397 | Down | protodermal factor 1 |
| LOC21385272 | | GTGATCCACAATTTCCGGATCCCATTTTATCT | Exon | 27.14 | 4.89 | -2.48918 | 2.08E-05 | Down | protein NPGR1 |
| LOC21385272 | | GGCTTTACAAGAGCCCGGCGATACGCAATAAT | Exon | 7.84 | 1.22 | -2.64208 | 0.012112 | Down | protein NPGR1 |
| LOC21390958 | | CATATCCAAACGTTCCGGCCACATCAGTGGTC | Exon | 6.03 | 0 | -6.34769 | 0.002947 | Down | LRR receptor-like serine/threonine-protein kinase RPK2 |
| LOC21397122 | | TGGCACAAGTGGCTCCGGAAAATCCACGGTTT | Exon | 3.02 | 10.4 | 1.724895 | 0.030086 | UP | ABC transporter B family member 15 |
| LOC21397122 | | AGGTATTCAATTATCCGGAGGACAAAAGCAAA | Intron | 0 | 7.95 | 6.706425 | 0.000489 | UP | ABC transporter B family member 15 |
| LOC21400775 | | GCGGAAAGGCGAGTCCGGCGAGCCCCGGGGAA | Exon | 0 | 3.67 | 5.606975 | 0.036027 | UP | aspartic proteinase nepenthesin-1 |
| LOC21400775 | | GAGTCCGGCGAGCCCCGGGGAATGAGCTTGAG | Exon | 0 | 6.12 | 6.332052 | 0.002947 | UP | aspartic proteinase nepenthesin-1 |
| LOC21388450 | | CAGCATGCTATTATCCGGCACGATCGCCAACG | Exon | 0 | 26.92 | 8.455651 | 9.21E-11 | UP | remorin 4.1 |
| LOC21396002 | | CCCAGGTAAGCATTCCGGTCATAGACCTCGGA | Exon | 0 | 23.25 | 8.244795 | 1.29E-09 | UP | 1-aminocyclopropane-1-carboxylate oxidase homolog 1 |
| LOC21401979 | | ATCATTTGGTCCCTCCGGCAGGGGACCATGTA | Exon | 45.24 | 12.24 | -1.91608 | 3.10E-05 | Down | monocopper oxidase-like protein SKU5 |
| LOC21401979 | | AACTTCTTACTCTACCGGTGATTTTGGCAATG | Intergenic | 27.14 | 11.01 | -1.33171 | 0.008884 | Down | monocopper oxidase-like protein SKU5 |
| LOC21389708 | | GGTTTCAAGAGTTTCCGGATTGGCTTTCCAAG | Exon | 7.24 | 0 | -6.60777 | 0.000883 | Down | disease resistance protein RPM1 |
| LOC21400231 | | GATGACGTCGTAGCCCGGAATGCCGCTGTTCG | Exon | 0 | 4.28 | 5.825133 | 0.019033 | UP | cationic peroxidase 2 |
| LOC21391131 | | AGGCCAAGAAACACCCGGCGAAGACCCCGTCC | Exon | 18.7 | 8.57 | -1.15556 | 0.045105 | Down | putative beta-D-xylosidase |
| LOC21394169 | | TTGCTCCGTTGCAGCCGGTAAAAGTTTGGTCT | Exon | 1.21 | 6.73 | 2.37299 | 0.031337 | UP | receptor-like protein kinase 5 |
| LOC21392066 | | TGCCCACGTGTCACCCGGGCAACAAGAAAGCG | Exon | 29.56 | 13.46 | -1.16655 | 0.01658 | Down | GEM-like protein 5 |
| LOC21400376 | | CTGGAACTCCATCACCGGAATTCGCTAAGTAT | Exon | 9.05 | 1.84 | -2.29046 | 0.013506 | Down | phosphomethylethanolamine N-methyltransferase |
| LOC21395936 | | CGATGTCTGTCAATCCGGGGAGATCGTTCTCA | Exon | 38 | 10.4 | -1.89787 | 8.77E-05 | Down | serine/threonine-protein kinase SAPK2 |
| LOC21408351 | | ACCTTGCCAACGCACCGGCAGTGCCACTCACC | Exon | 0 | 3.67 | 5.606975 | 0.036027 | UP | CBS domain-containing protein CBSX5 |
| LOC21394858 | | ATCATGTTCTACATCCGGGACATCTTTTCGAA | Exon | 25.33 | 1.84 | -3.76824 | 1.60E-07 | Down | uncharacterized LOC21394858 |
| LOC21386721 | | AGGCTAATTGACAGCCGGAAACTATCCCCGGA | Exon | 4.22 | 0 | -5.84069 | 0.019033 | Down | root phototropism protein 3 |
| LOC21397198 | | CGACTCAAAGACGACCGGTTGCAGCAAAACAA | Exon | 2.41 | 14.68 | 2.532508 | 0.000829 | UP | rust resistance kinase Lr10 |
| LOC21410440 | | ATCTCAGCCGTGCACCGGTGCACCGCCGCTTG | Exon | 3.62 | 0 | -5.62249 | 0.036027 | Down | IAA-amino acid hydrolase ILR1-like 2 |
| LOC21410270 | | ATCCATAAACCTCTCCGGGATGAATTCCTCGG | Exon | 22.92 | 11.01 | -1.08851 | 0.039801 | Down | cytochrome P450 71B37 |
| LOC21387864 | | TATTTCACATACCTCCGGCATTGAGAGATTCG | Intron | 4.83 | 20.8 | 2.054705 | 0.000736 | UP | glutamate receptor 2.8 |
| LOC21407221 | | GGGCTTTTTTAGCTCCGGAGACTCCAATGCCG | Exon | 36.79 | 17.13 | -1.13569 | 0.012177 | Down | uncharacterized LOC21407221 |
| LOC21393642 | | GTCACTTCCGGCGGCCGGGCTGCTCGCGGAGT | Exon | 19.9 | 1.22 | -3.97773 | 1.22E-06 | Down | F-box/LRR-repeat MAX2 homolog A |
| LOC21387791 | | CGTGACCTATATTGCCGGTCCTAACATAACTT | Intron | 41.62 | 99.12 | 1.214012 | 0.000686 | UP | alpha-galactosidase |
| LOC21394538 | | TGCTGGTTGAAGATCCGGTGACCGACGATGTC | Exon | 0 | 21.41 | 8.126558 | 5.10E-09 | UP | heat stress transcription factor B-3 |
| LOC21407908 | | TGAGGACAAAACGACCGGACAAAAGAACAAGA | Exon | 3.02 | 11.01 | 1.806769 | 0.020789 | UP | heat shock cognate 70 kDa protein 2 |
| LOC21402514 | | TATTTCTACTATTGCCGGGAGCCGCCGGCGAT | Exon | 7.24 | 17.74 | 1.248553 | 0.032805 | UP | glucan endo-1,3-beta-glucosidase 5 |
| LOC21410042 | | ATAGCCTCGCATATCCGGCGCAACGACACGAA | Exon | 3.02 | 18.35 | 2.539728 | 0.000219 | UP | epoxide hydrolase A |
| LOC21410115 | | AGCGGAGGAAGAGACCGGAGACGCGATGACGA | Exon | 10.86 | 25.09 | 1.166278 | 0.021153 | UP | O-acyltransferase WSD1 |
| LOC21393677 | | ATTCAGGCCAAATTCCGGTGTCGAACACCCCT | Exon | 2.41 | 9.79 | 1.951299 | 0.021106 | UP | cucumisin |
| LOC21393969 | | AGTCAGTTGCTACACCGGTCGTTGGTCGAGGA | Exon | 1.81 | 24.47 | 3.667079 | 3.50E-07 | UP | probable serine/threonine-protein kinase WNK4 |
| LOC21399822 | | ATCCATCTCGCTGCCCGGCGGGTAAGCGCACC | Exon | 8.44 | 1.22 | -2.74802 | 0.007516 | Down | cytochrome P450 77A4 |
| LOC21406110 | | AAGGGGAAGCAGAACCGGAAAGCCGGCCGATT | Exon | 0 | 7.95 | 6.706425 | 0.000489 | UP | probable purine permease 11 |
