# Supplementary material for: Transcriptome and DNA Methylome Reveal Insights Into Phytoplasma Infection Responses in Mulberry (Morus multicaulis Perr.)
Source: Front Plant Sci. 2021 Aug 3;12:697702. doi: 10.3389/fpls.2021.697702 (PMC8369481; doi:10.3389/fpls.2021.697702)
Supplement: Supplementary Table 7 — Differentially methylated levels in CCWGG sites in the gene regions of differential methylated and expressed genes in the healthy and infected mulberry leaves. [file Table_7.DOCX]

**Table S7. Differential methylated levels in CCWGG sites in the gene regions of differential methylated and expressed genes in the healthy and infected mulberry leaves.**

| **Gene id** | **Sequence** | **Gene**  **regions** | **Normalized value** | | **Fold-change** | **P-Value** | **Up/**  **down** | **Description** |
| --- | --- | --- | --- | --- | --- | --- | --- | --- |
|  |  |  | **IL** | **HL** | **log2(IL/HL)** |  |  |  |
| LOC21386217 | TGGTTTGTCTCCACCGGGGCGAGCGGAAATG | Exon | 4.15 | 23.11 | -2.459300179 | 0.008739612 | Down | GATA transcription factor 8 |
| LOC21405760 | CTCTATTGTGGGACCAGGTACTGTTGCGGCT | Exon | 76.08 | 26.19 | 1.498665887 | 0.001861415 | UP | multicopper oxidase LPR1 |
| LOC21406640 | ATTATTCTCGTCACCAGGTAATTATTTAAGC | Exon | 8.3 | 0 | 5.525300744 | 0.036026696 | UP | protein ECERIFERUM 1 |
| LOC21408270 | TCTTCCCCCCACTCCCGGTACTCTTCTCCGC | Exon | 8.3 | 0 | 5.525300744 | 0.036026696 | UP | UDP-glucuronate 4-epimerase 3 |
| LOC21386728 | AGAGCAAGAGTCACCCGGCTGCTTTGACGAC | Exon | 17.98 | 1.54 | 3.366893481 | 0.005616358 | UP | pleiotropic drug resistance protein 1 |
| LOC21385193 | TAACTCTCCACCACCAGGTGAGTCTTTAAGG | Exon | 2.77 | 46.21 | -4.008338712 | 1.89E-06 | Down | uncharacterized LOC21385193 |
| LOC21397798 | GGATCCTGCACTTCCCGGAGCAGAAATTCGA | Exon | 12.45 | 0 | 6.099783555 | 0.00544505 | UP | alpha-xylosidase 1 |
| LOC21408202 | AAGATAGCAGTTTCCCGGCATCTGCTTTTAC | Exon | 1.38 | 26.19 | -4.105819488 | 0.000243198 | Down | non-lysosomal glucosylceramidase |
| LOC21385834 | CTGTAATGACAGCCCCGGAGCAAAGTTCATC | Exon | 22.13 | 0 | 6.920625961 | 0.000153075 | UP | uncharacterized acetyltransferase At3g50280 |
| LOC21409371 | GGCTTAAAACTATCCGGGTCGGCCCACGAGT | Exon | 8.3 | 0 | 5.525300744 | 0.036026696 | UP | cytochrome P450 84A1 |
| LOC21383997 | ACTCAGAGTGATGCCTGGACTAGTTATGGAT | Exon | 3 | 8.7 | -1.5360529 | 0.020021665 | Down | G-type lectin S-receptor-like serine/threonine-protein kinase |
