# Supplementary figures and images for: Transcriptome and DNA Methylome Reveal Insights Into Phytoplasma Infection Responses in Mulberry (Morus multicaulis Perr.)
Source: Front Plant Sci. 2021 Aug 3;12:697702. doi: 10.3389/fpls.2021.697702 (PMC8369481; doi:10.3389/fpls.2021.697702)

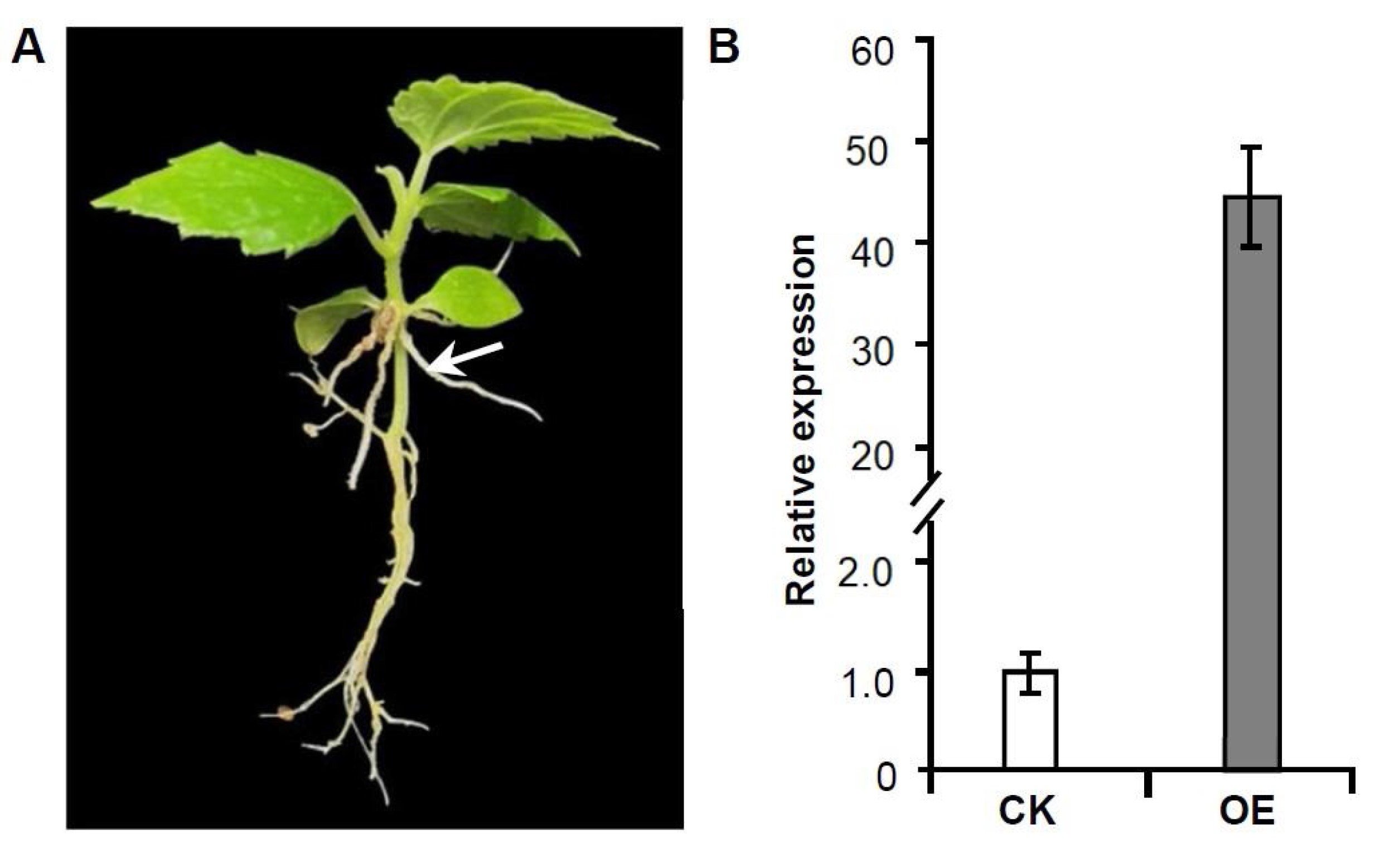

Supplement: Supplementary Figure 1 — Regeneration of transgenic Mu-GsSRK hairy roots in mulberry seedling. (A) The hairy root phenotype of mulberry seedling. The arrow points to the hairy root. (B) Quantitative analysis of the expression levels of the Mu-GsSRK genes in the transgenic hairy roots. The relative expression levels of the genes were evaluated using the 2−ΔΔCt method with the Mul-actin and Mul-EF1-α as reference genes. Data represent the mean values of triplicate samples ± SD. Asterisk indicates significant difference at P < 0.05 between WT and OE plants according to Student's t-test. WT, wild type plants; CK, the transgenic empty vector hairy roots; OE, the transgenic Mu-GsSRK hairy roots. [file Image_1.JPEG]
